# Supplementary material for: Five New Pregnane Glycosides from Gymnema sylvestre and Their α-Glucosidase and α-Amylase Inhibitory Activities
Source: Molecules. 2020 May 28;25(11):2525. doi: 10.3390/molecules25112525 (PMC7321224; doi:10.3390/molecules25112525)

molecules

Supplementary Material

Five New Pregnane Glycosides from *Gymnema sylvestre* and Their α-Glucosidase and α-Amylase Inhibitory Activities

Phan Van Kiem [1,2], Duong Thi Hai Yen [1,2], Nguyen Van Hung [1], Nguyen Xuan Nhiem [1,2], Bui Huu Tai [1,2], Do Thi Trang [1], Pham Hai Yen [1], Tran Minh Ngoc [3], Chau Van Minh [1], SeonJu Park [4], Jae Hyuk Lee [5], Sun Yeou Kim [6,7] and Seung Hyun Kim [6,*]

[1] Institute of Marine Biochemistry, Vietnam Academy of Science and Technology (VAST), 18 Hoang Quoc Viet, Cau Giay, Hanoi zip code, Vietnam; phankiem@yahoo.com (P.V.K.), haikienk51a@gmail.com (D.T.H.Y.), hungnvd8@yahoo.com (N.V.H.), nxnhiem@yahoo.com (N.X.N.), bhtaiich@gmail.com (B.H.T.), trang2002.imbc@gmail.com (D.T.T.), yeninpc@yahoo.com (P.H.Y.), cvminh@vast.vn (C.V.M.)

[2] Department, Graduate University of Science and Technology, VAST, 18 Hoang Quoc Viet, Cau Giay, Hanoi zip code, Vietnam

[3] Traditional Medicine Administration, Ministry of Health, 138A Giang Vo, Ba Dinh, Hanoi zip code, Vietnam; tmngocvkn@gmail.com

[4] Chuncheon Center, Korea Basic Science Institute (KBSI), Chuncheon 24341, Korea; sjp19@kbsi.re.kr

[5] Department, College of Pharmacy, Gachon University, 191, Hambakmoero, Yeonsu-gu, Incheon 21936, Korea; wogur6378@naver.com (J.H.L.), sunnykim@gachon.ac.kr (S.Y.K.)

[6] Yonsei Institute of Pharmaceutical Science, College of Pharmacy, Yonsei University, Incheon 21983, Korea

[7] Gachon Institute of Pharmaceutical Science, Gachon University, 191, Hambakmoero, Yeonsu-gu, Incheon 21936, Korea

* Correspondence: kimsh11@yonsei.ac.kr

Content

Figure S1: The chemical structures of compounds **6-9** 1

Figure S2: HR-ESI-MS of compound **1** 1

Figure S3: ^{1}H-NMR spectrum of compound **1** 2

Figure S4: ^{13}C-NMR spectrum of compound **1** 3

Figure S5: DEPT135 spectrum of compound **1** 4

Figure S6: HSQC spectrum of compound **1** 5

Figure S7: HMBC spectrum of compound **1** 6

Figure S8: COSY spectrum of compound **1** 7

Figure S9: ROESY spectrum of compound **1** 8

Figure S10: HR-ESI-MS of compound **2** 8

Figure S11: ^{1}H-NMR spectrum of compound **2** 9

Figure S12: ^{13}C-NMR spectrum of compound **2** 9

Figure S13: DEPT135 spectrum of compound **2** 10

Figure S14: HSQC spectrum of compound **2** 11

Figure S15: HMBC spectrum of compound **2** 12

Figure S16: COSY spectrum of compound **2** 13

Figure S17: ROESY spectrum of compound **2** 14

Figure S18: HR-ESI-MS of compound **3** 14

Figure S19: ^{1}H-NMR spectrum of compound **3** 15

Figure S20: ^{13}C-NMR spectrum of compound **3** 16

Figure S21: HSQC spectrum of compound **3** 17

Figure S22: HMBC spectrum of compound **3** 18

Figure S23: COSY spectrum of compound **3** 19

Figure S24: ROESY spectrum of compound **3** 20

Figure S25: HR-ESI-MS of compound **4** 20

Figure S26: ^{1}H-NMR spectrum of compound **4** 21

Figure S27: ^{13}C-NMR spectrum of compound **4** 22

Figure S28: DEPT135 spectrum of compound **4** 23
Figure S29: HSQC spectrum of compound **4** 24
Figure S30: HMBC spectrum of compound **4** 25
Figure S31: COSY spectrum of compound **4** 26
Figure S32: ROESY spectrum of compound **4** 27
Figure S33: HR-ESI-MS of compound **5** 27
Figure S34: ^{1}H-NMR spectrum of compound **5** 28
Figure S35: ^{13}C-NMR spectrum of compound **5** 29
Figure S36: DEPT135 spectrum of compound **5** 30
Figure S37: HSQC spectrum of compound **5** 31
Figure S38: HMBC spectrum of compound **5** 32
Figure S39: COSY spectrum of compound **5** 33
Figure S40: ^{1}H-NMR spectrum of compound **6** 34
Figure S41: ^{13}C-NMR spectrum of compound **6** 35
Figure S42: HSQC spectrum of compound **6** 36
Figure S43: ^{1}H-NMR spectrum of compound **7** 37
Figure S44: ^{13}C-NMR spectrum of compound **7** 38
Figure S45: HSQC spectrum of compound **7** 39
Figure S46: ^{1}H-NMR spectrum of compound **8** 40
Figure S47: ^{13}C-NMR spectrum of compound **8** 41
Figure S48: HSQC spectrum of compound **8** 42
Figure S49: ^{1}H-NMR spectrum of compound **9** 43
Figure S50: ^{13}C-NMR spectrum of compound **9** 44
Figure S51: HSQC spectrum of compound **9** 45

Figure S1: The chemical structures of compounds **6-9**

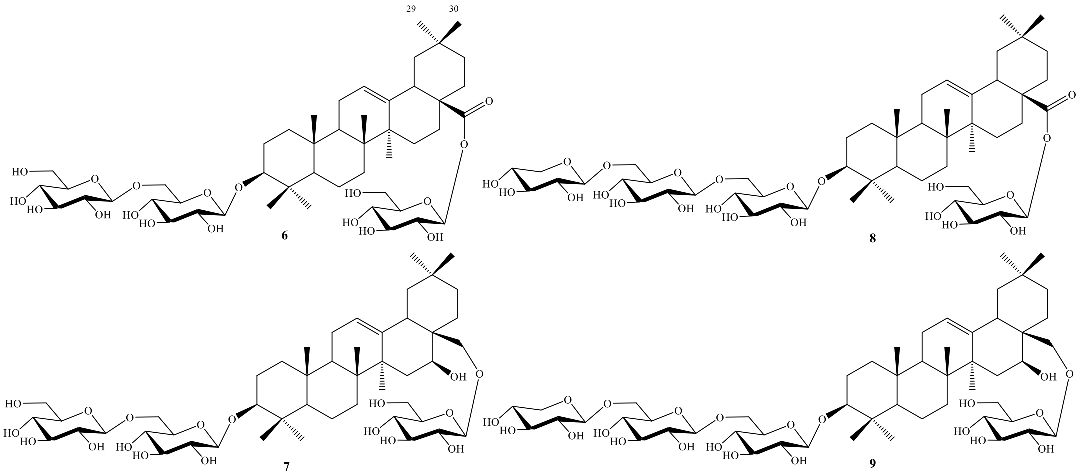

Figure S2: HR-ESI-MS of compound **1**

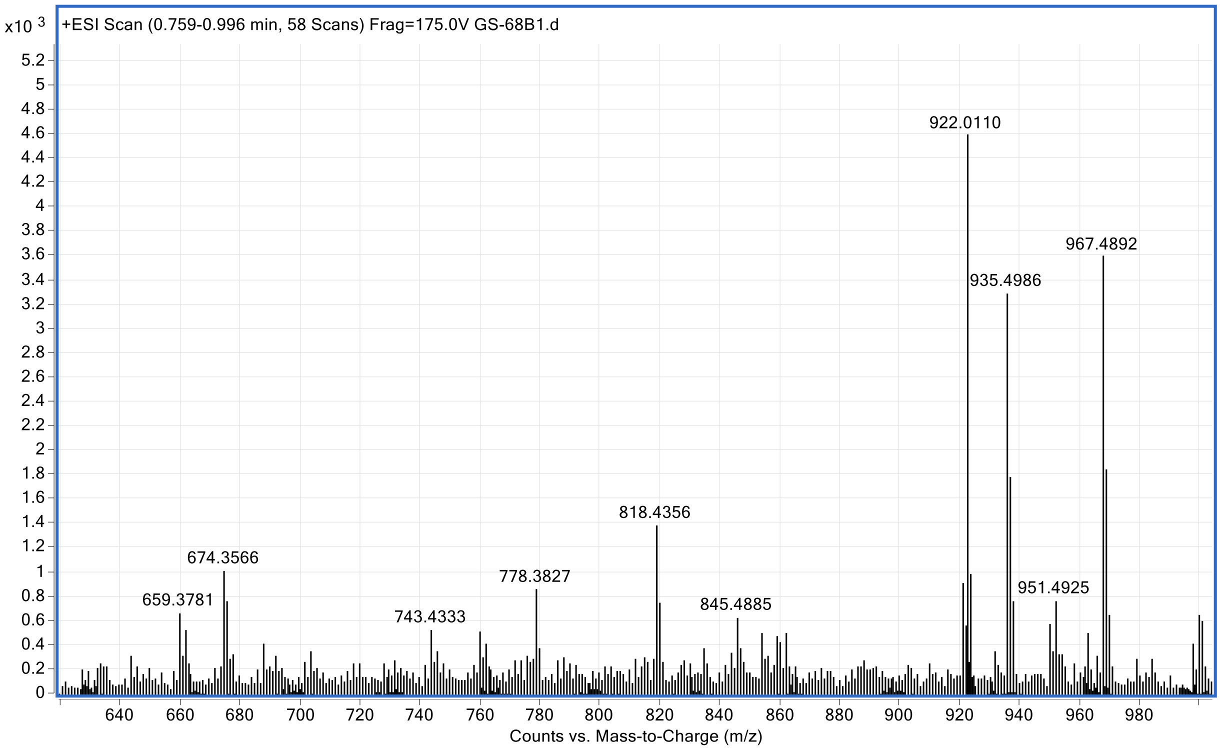

Figure S3: ^{1}H-NMR spectrum of compound **1**

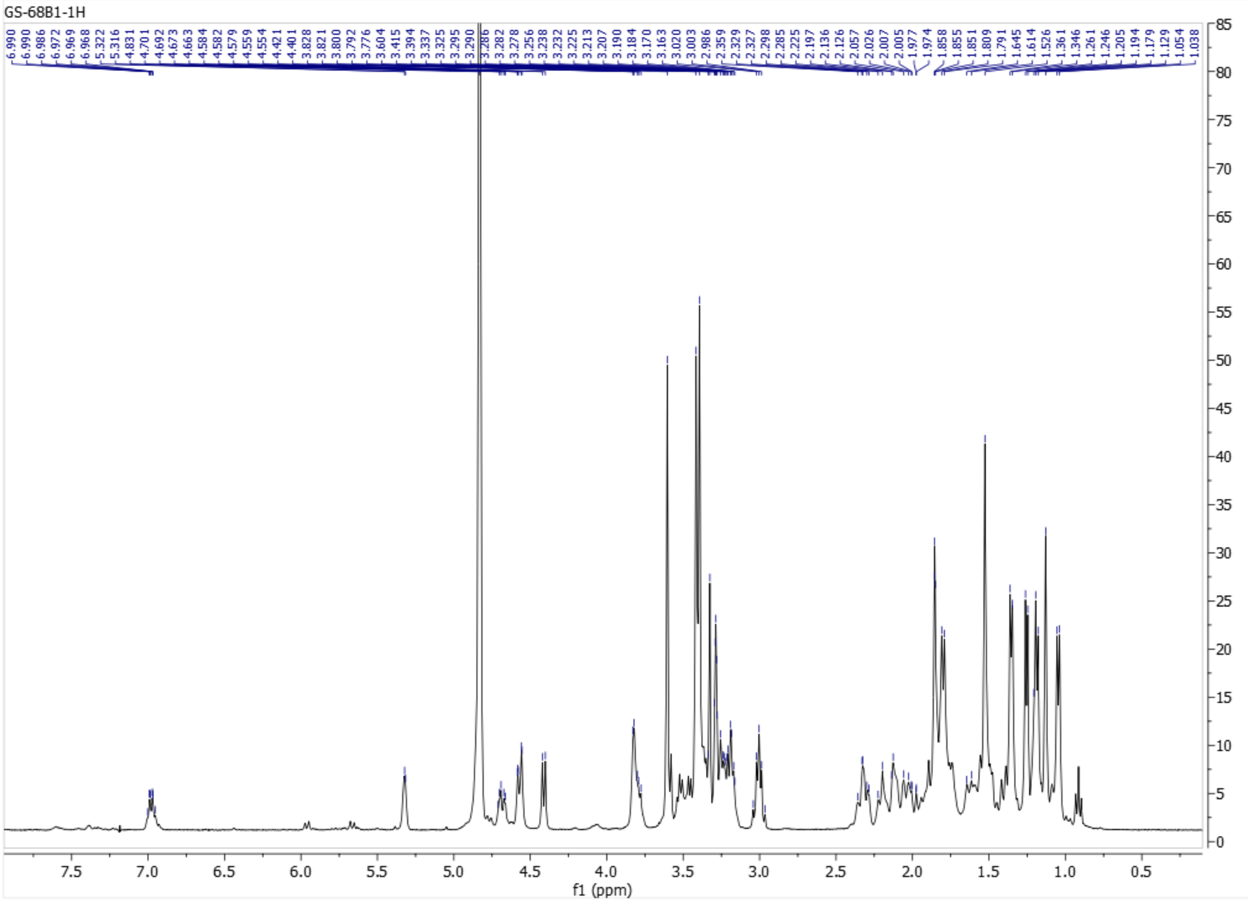

Figure S4: ^{13}C-NMR spectrum of compound **1**

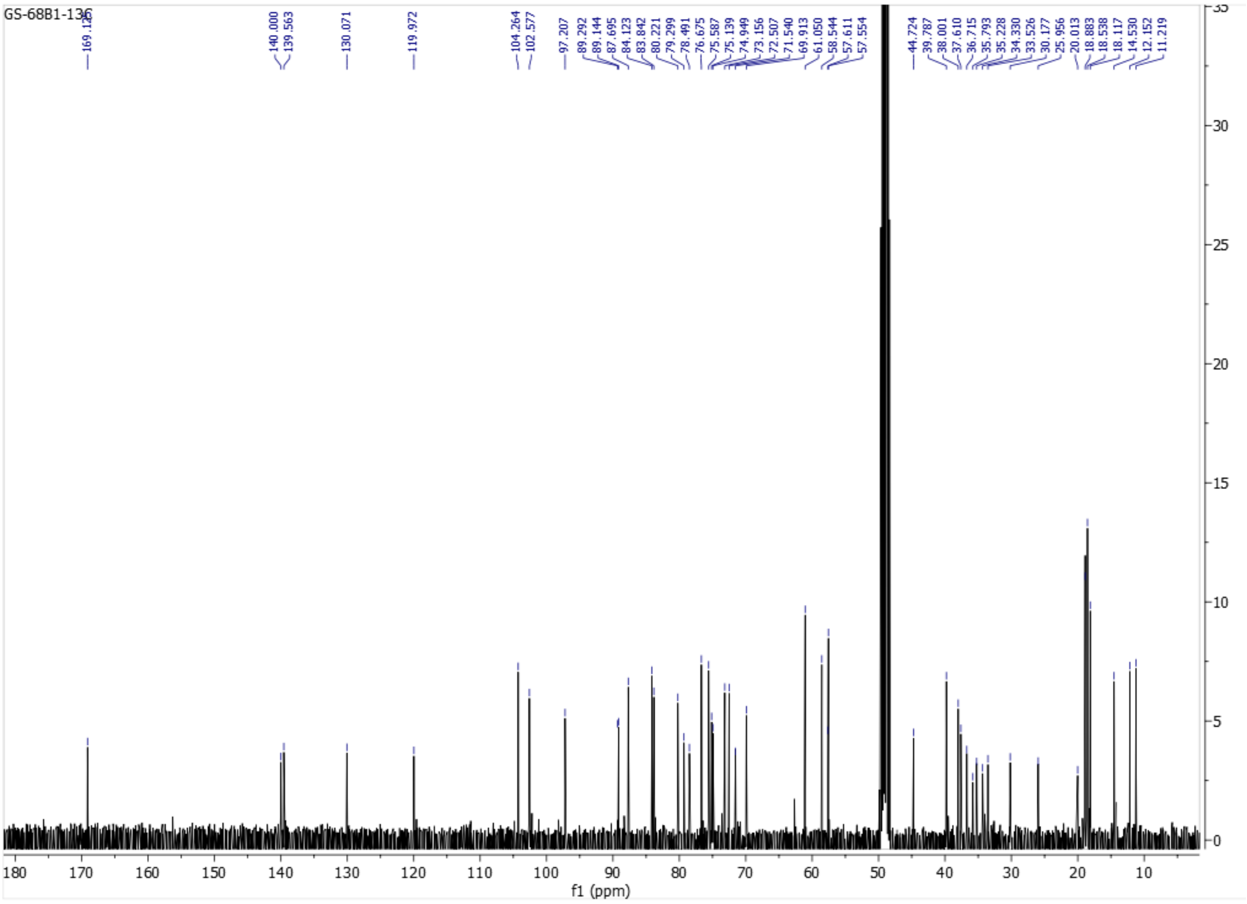

Figure S5: DEPT135 spectrum of compound **1**

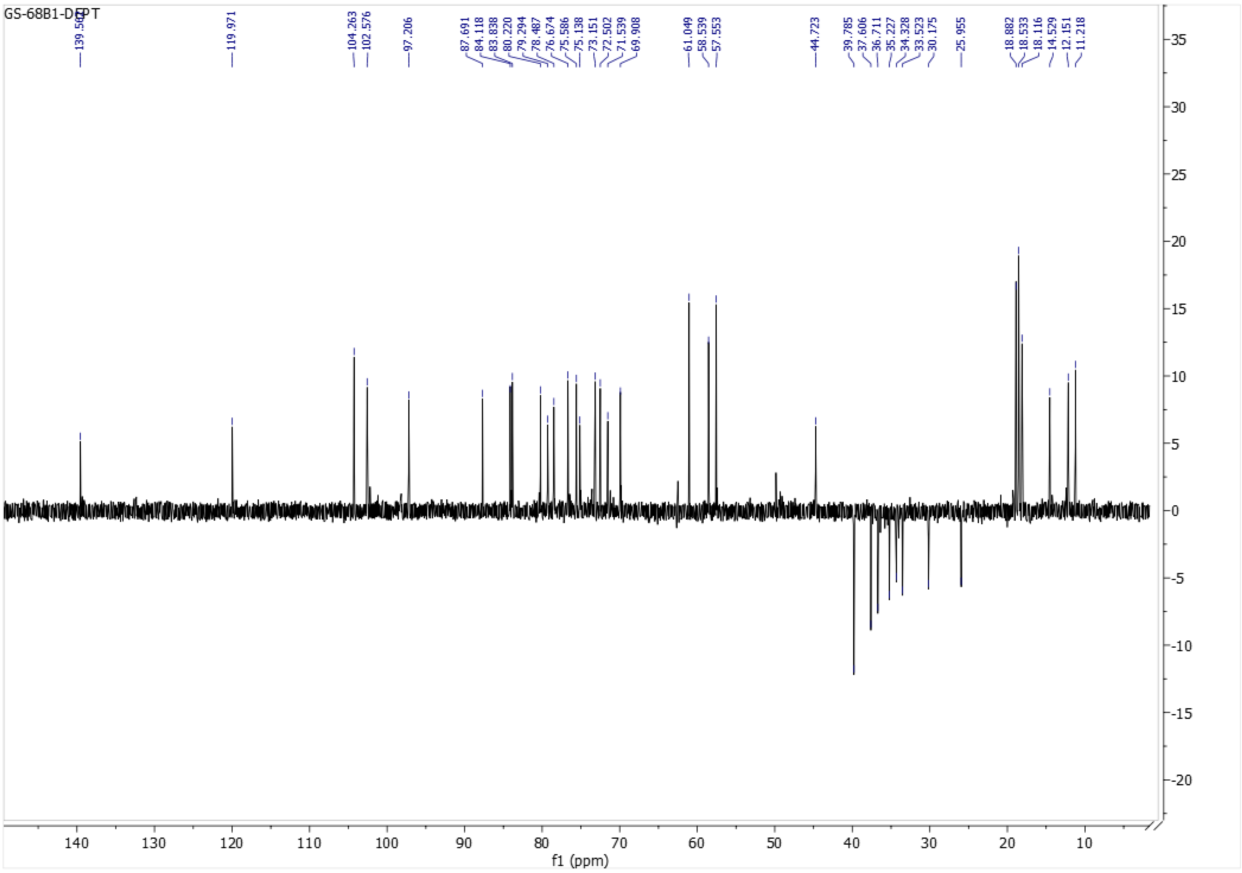

Figure S6: HSQC spectrum of compound **1**

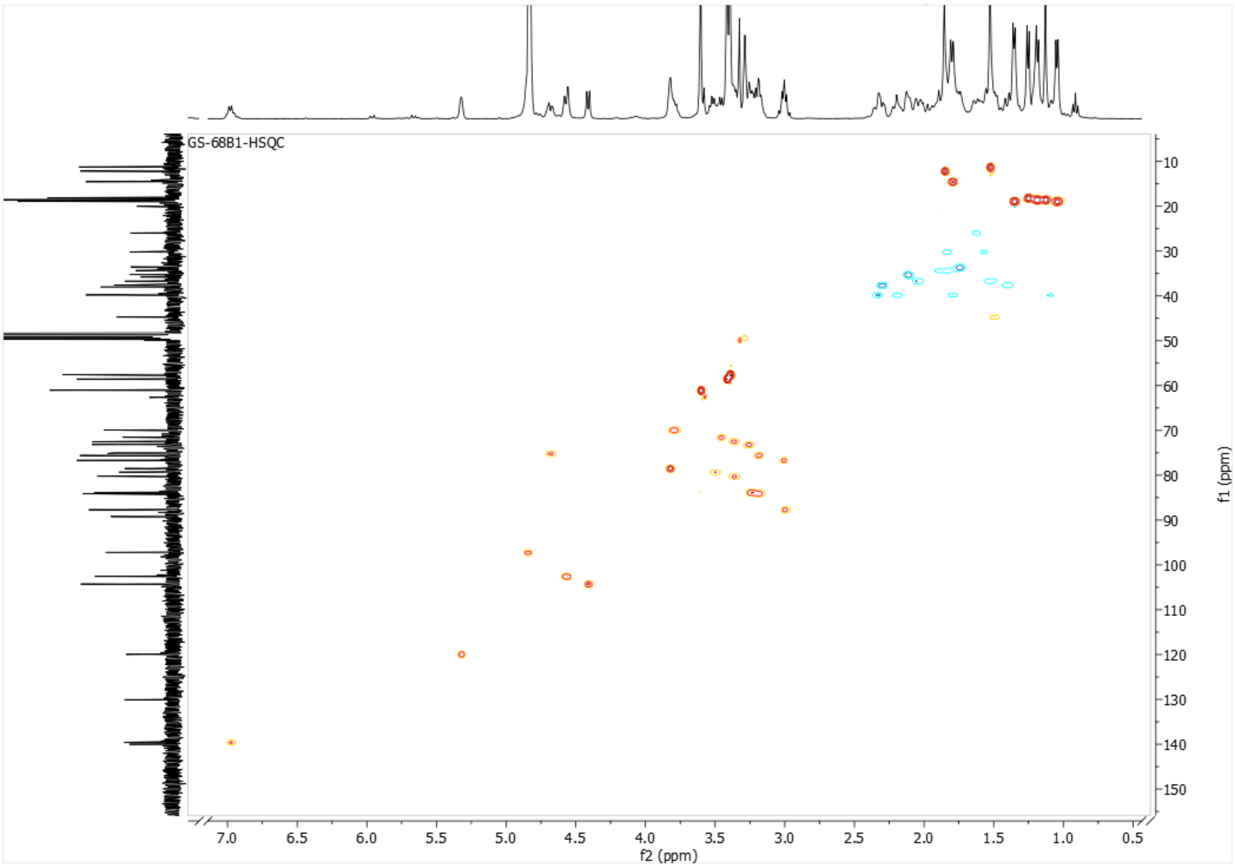

Figure S7: HMBC spectrum of compound **1**

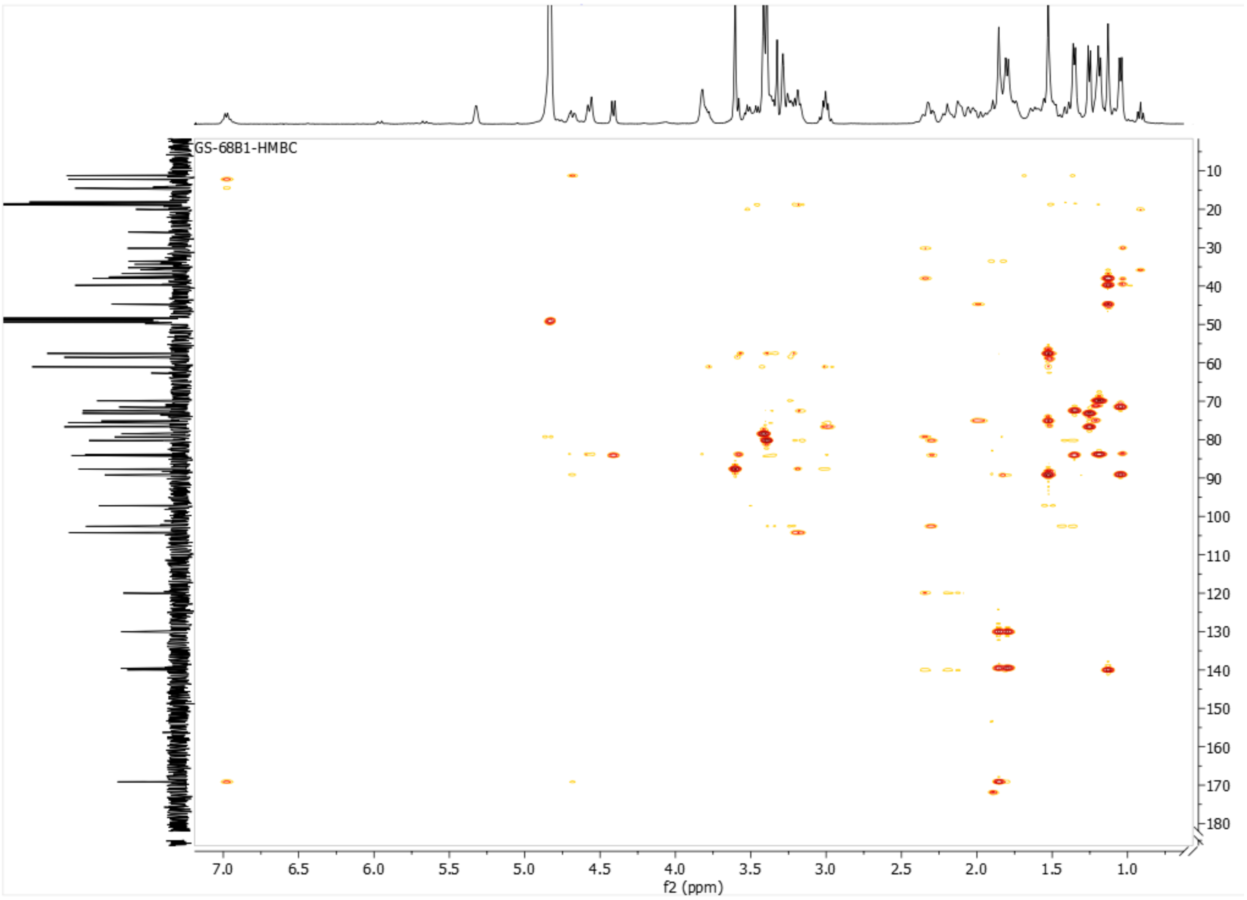

Figure S8: COSY spectrum of compound **1**

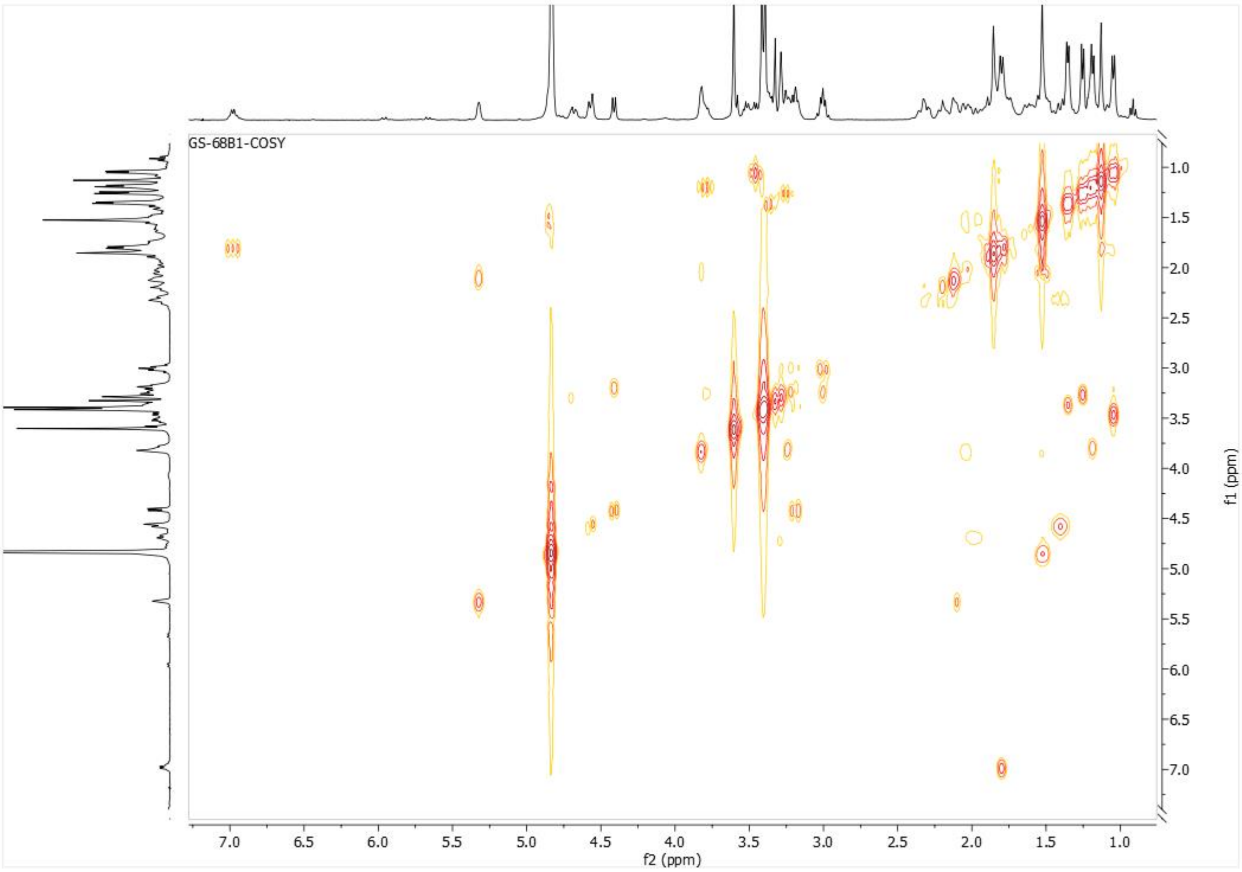

Figure S9: ROESY spectrum of compound **1**

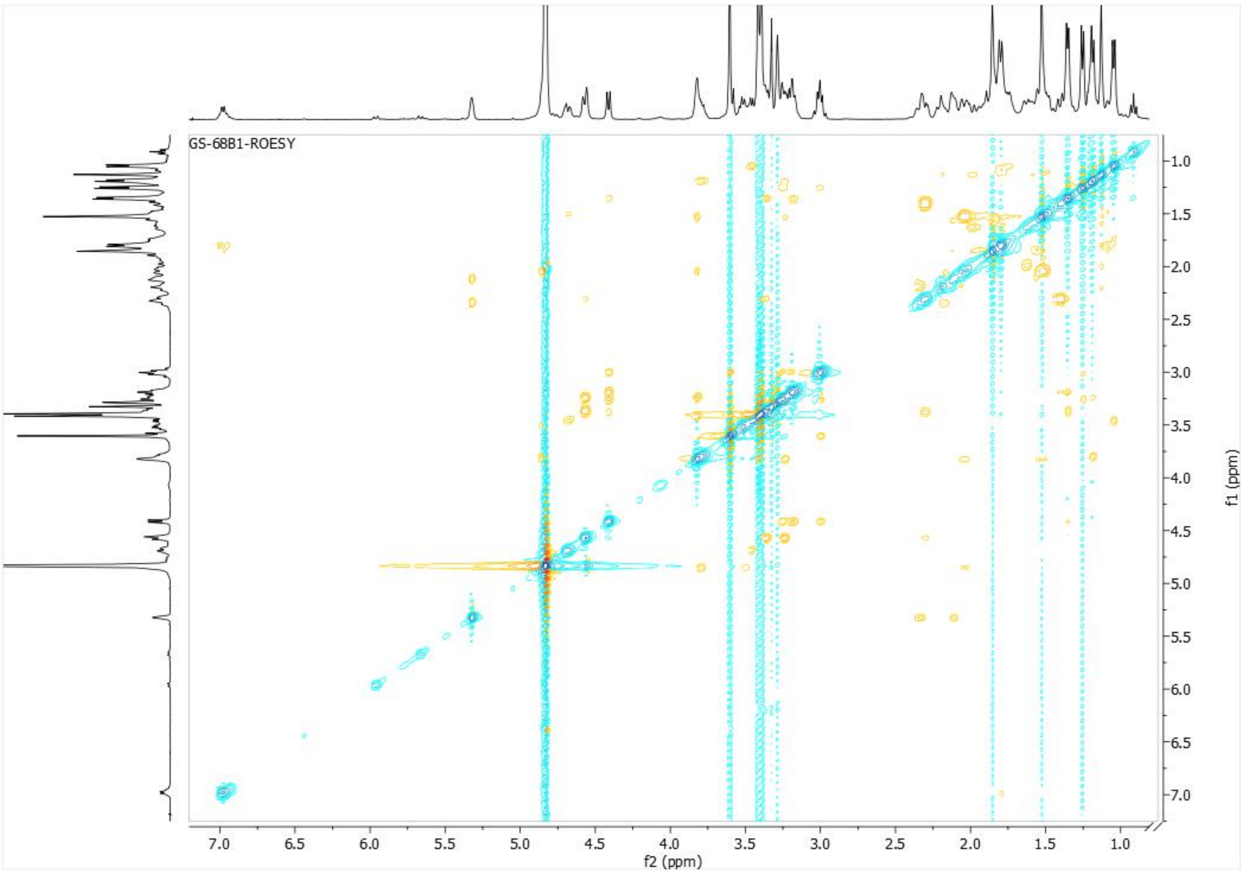

Figure S10: HR-ESI-MS of compound **2**

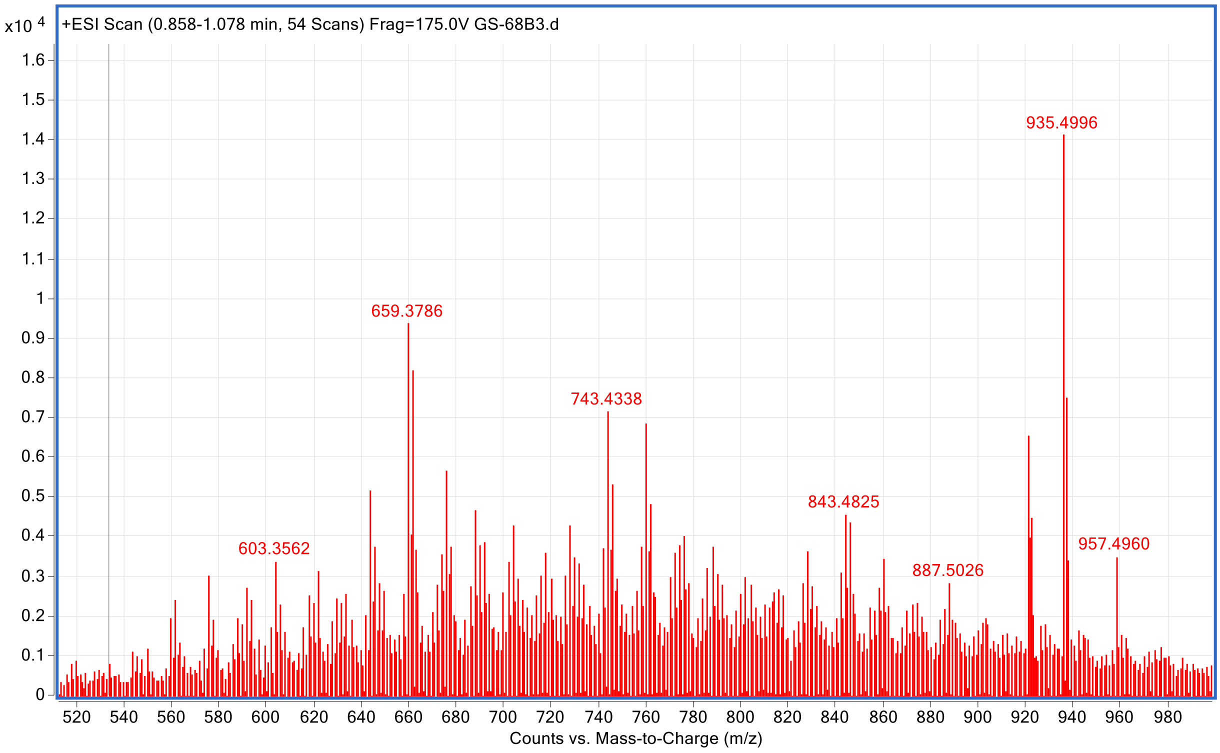

Figure S11: ^{1}H-NMR spectrum of compound **2**

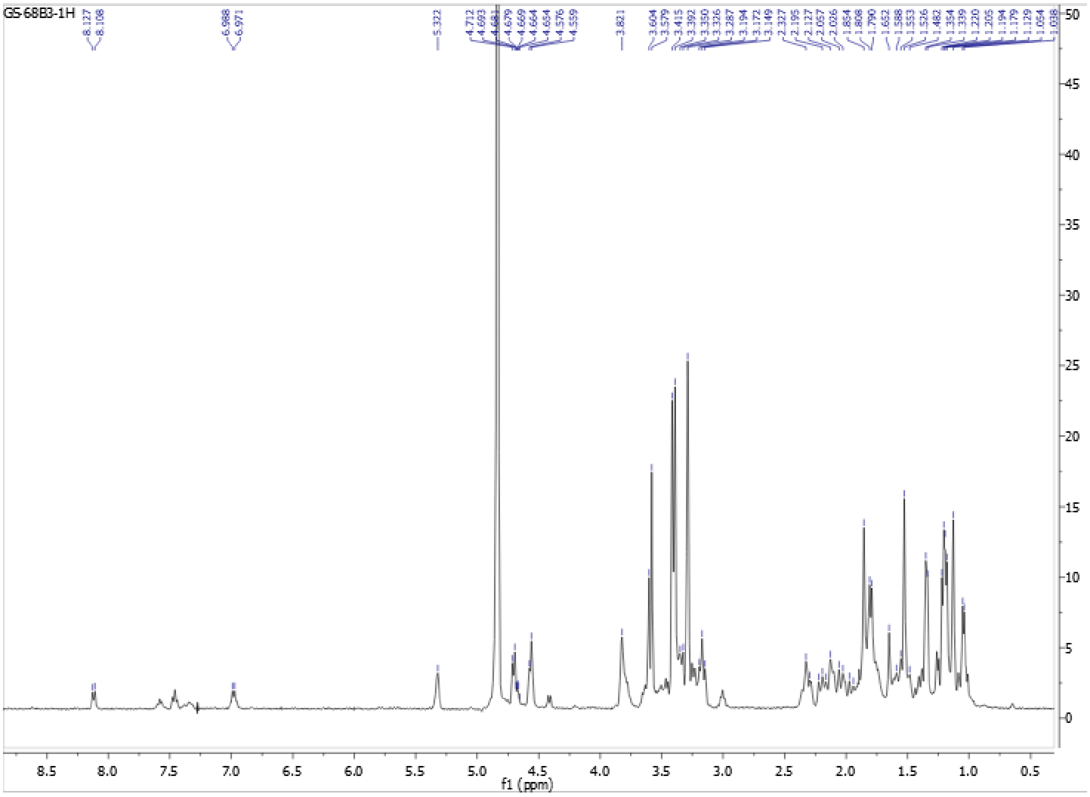

Figure S12: ^{13}C-NMR spectrum of compound **2**

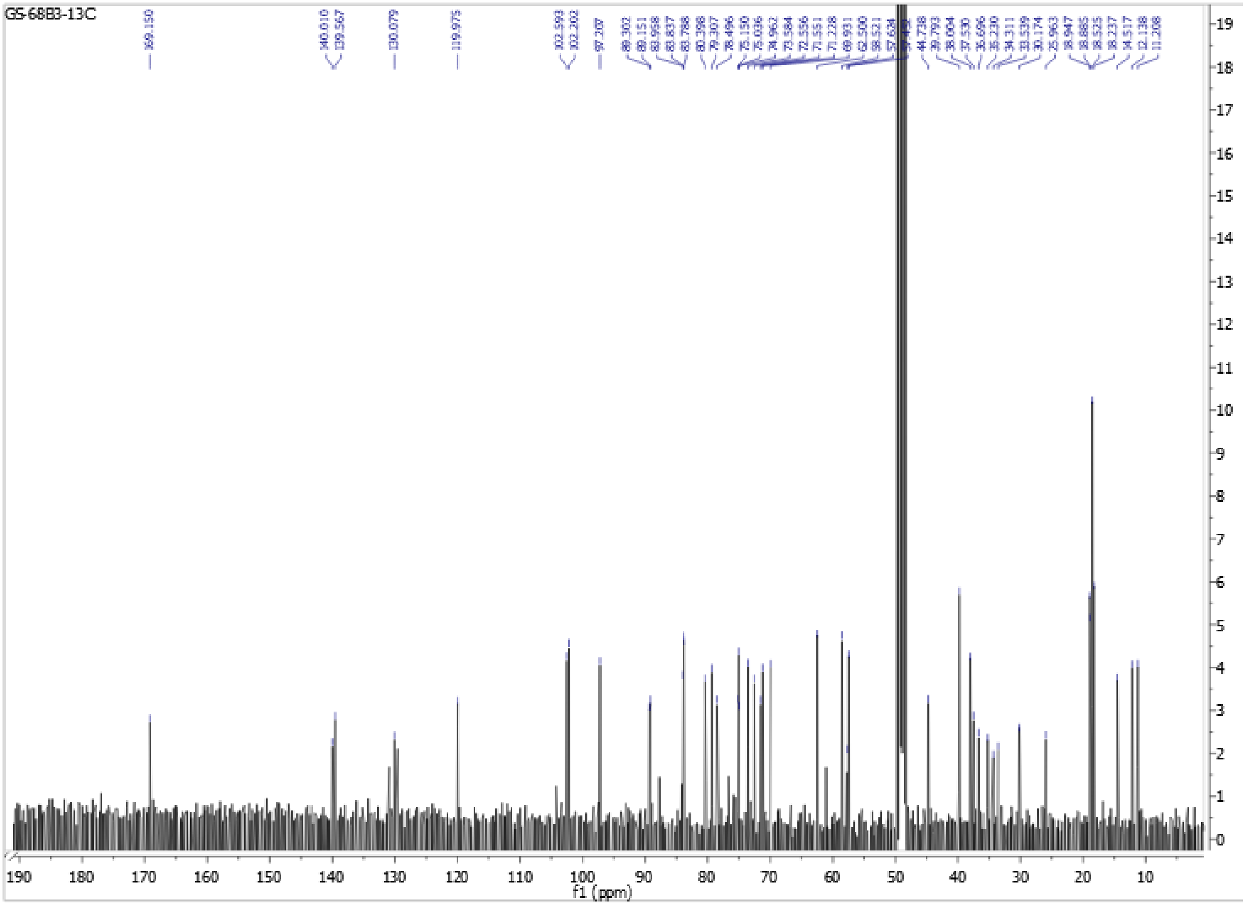

Figure S13: DEPT135 spectrum of compound **2**

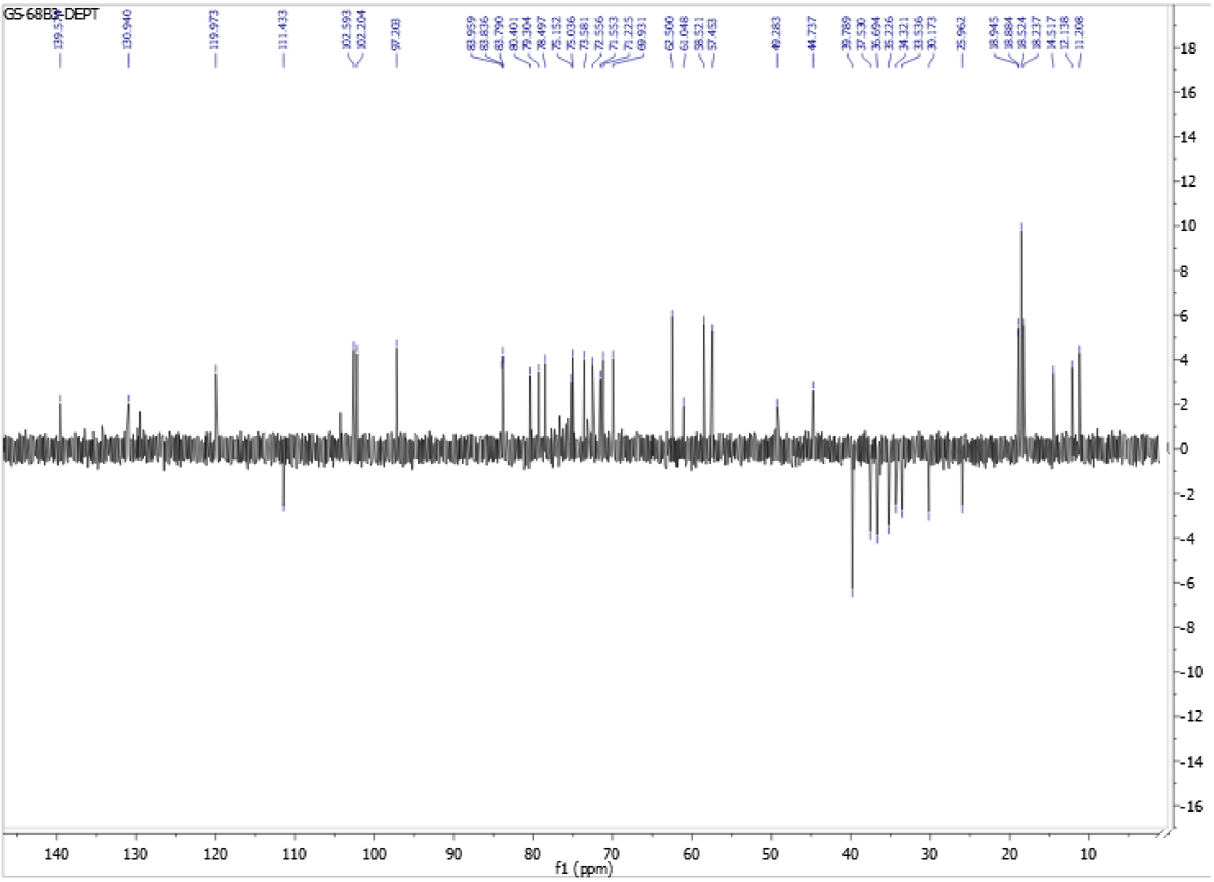

Figure S14: HSQC spectrum of compound **2**

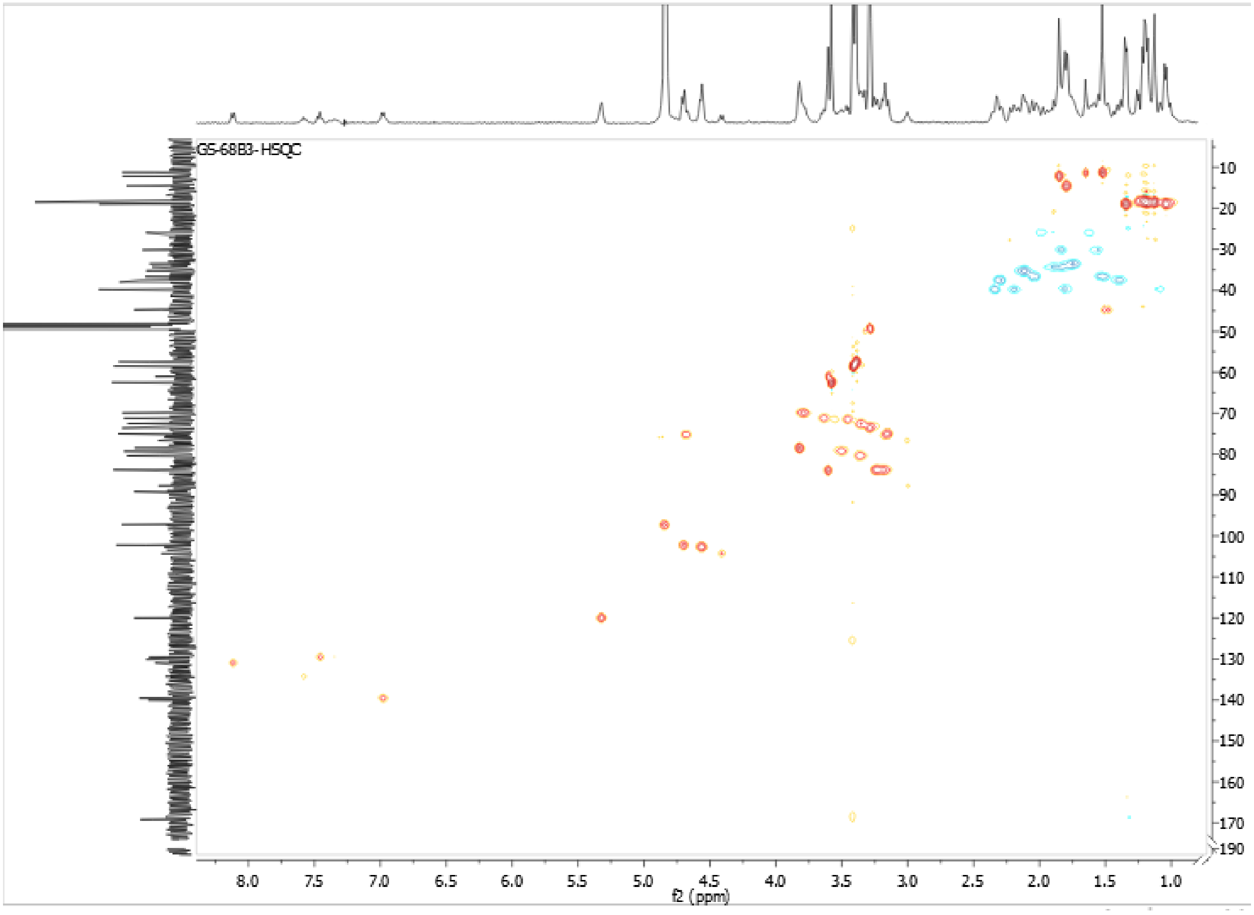

Figure S15: HMBC spectrum of compound **2**

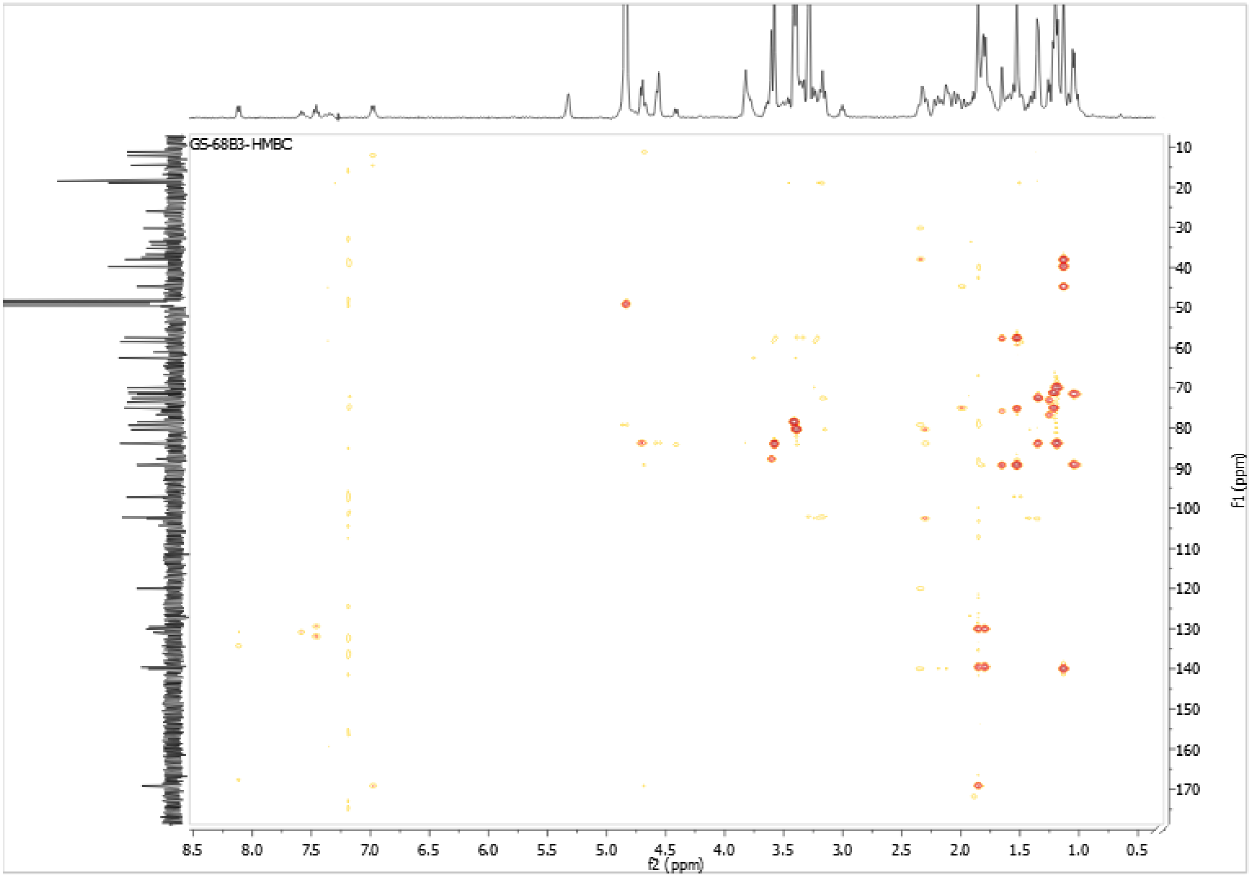

Figure S16: COSY spectrum of compound **2**

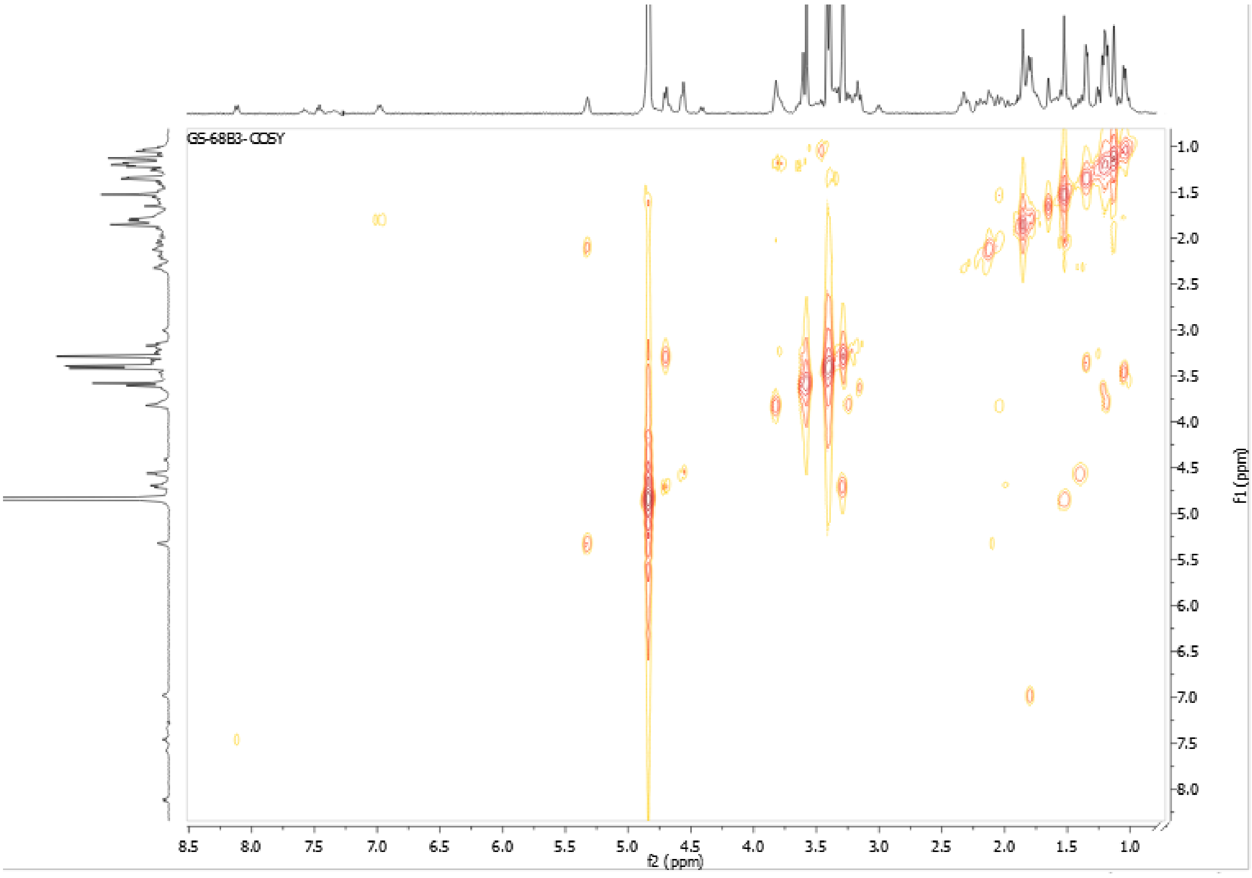

Figure S17: ROESY spectrum of compound **2**

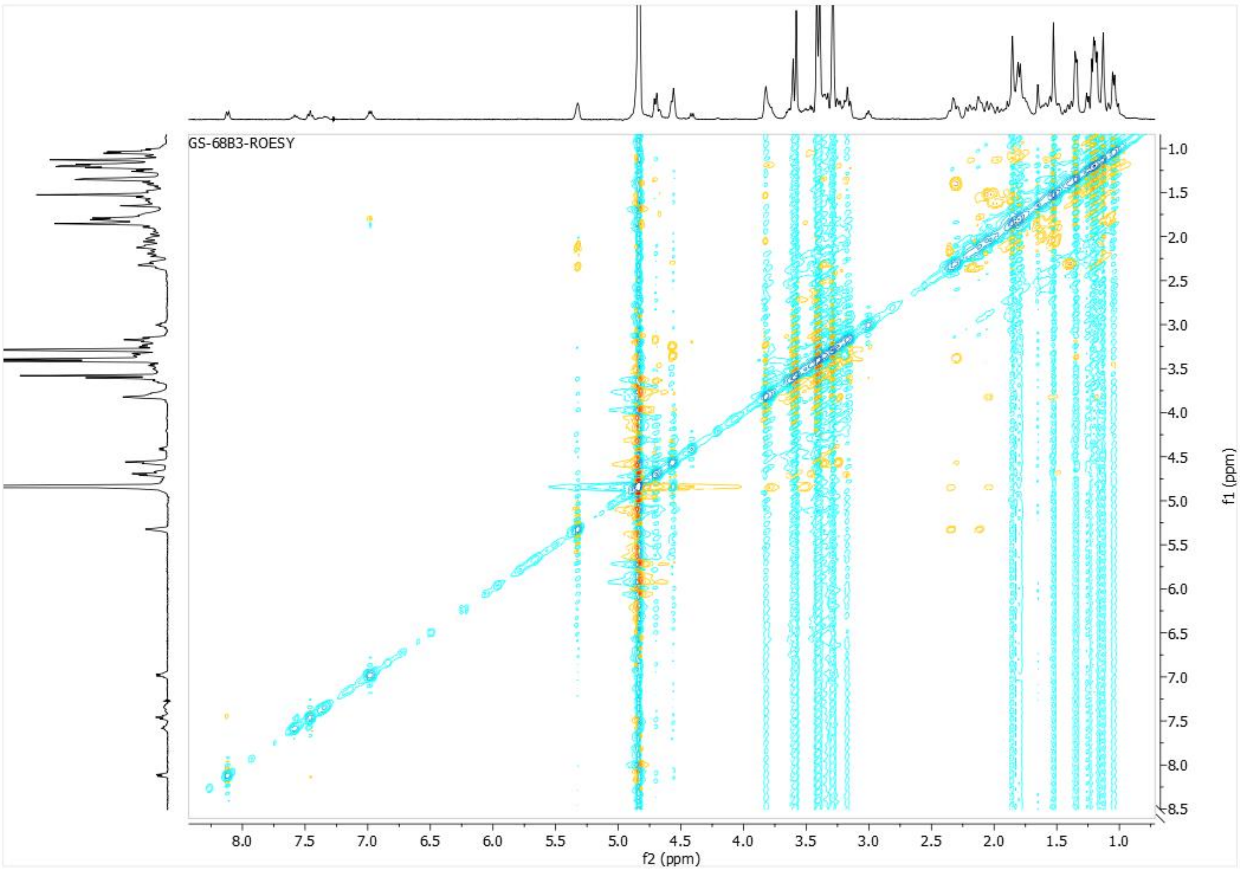

Figure S18: HR-ESI-MS of compound **3**

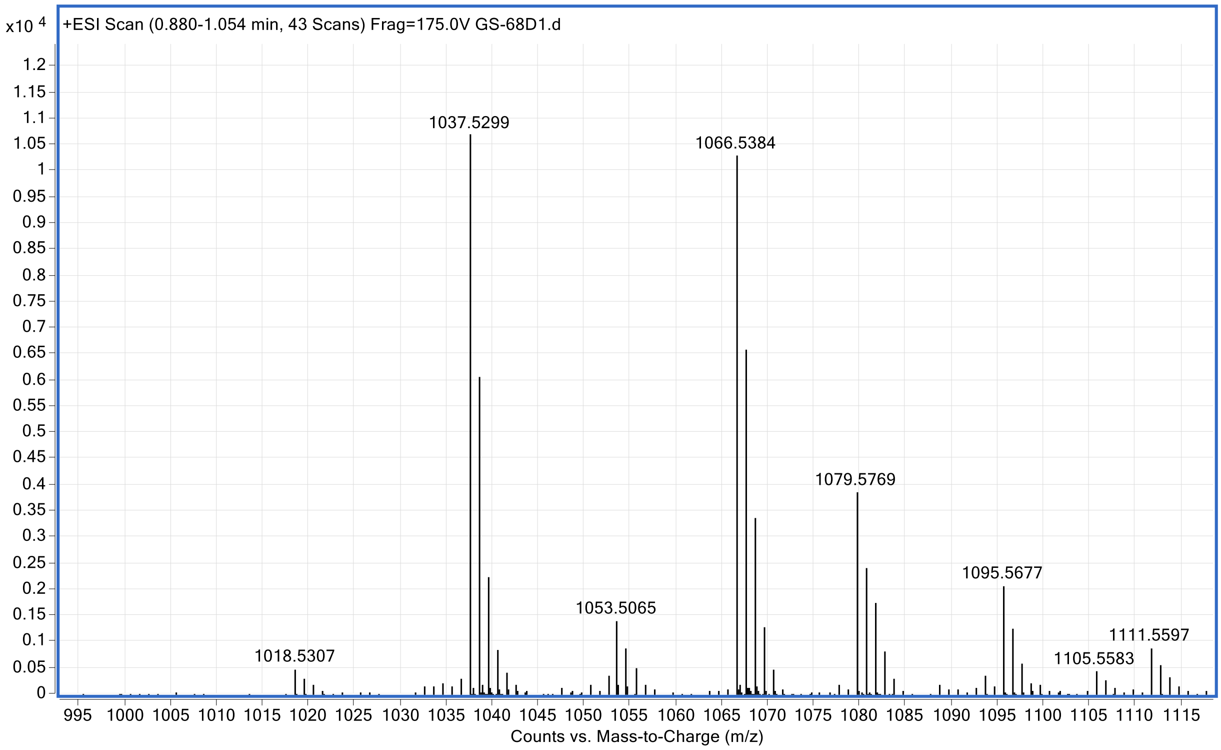

Figure S19: ^{1}H-NMR spectrum of compound **3**

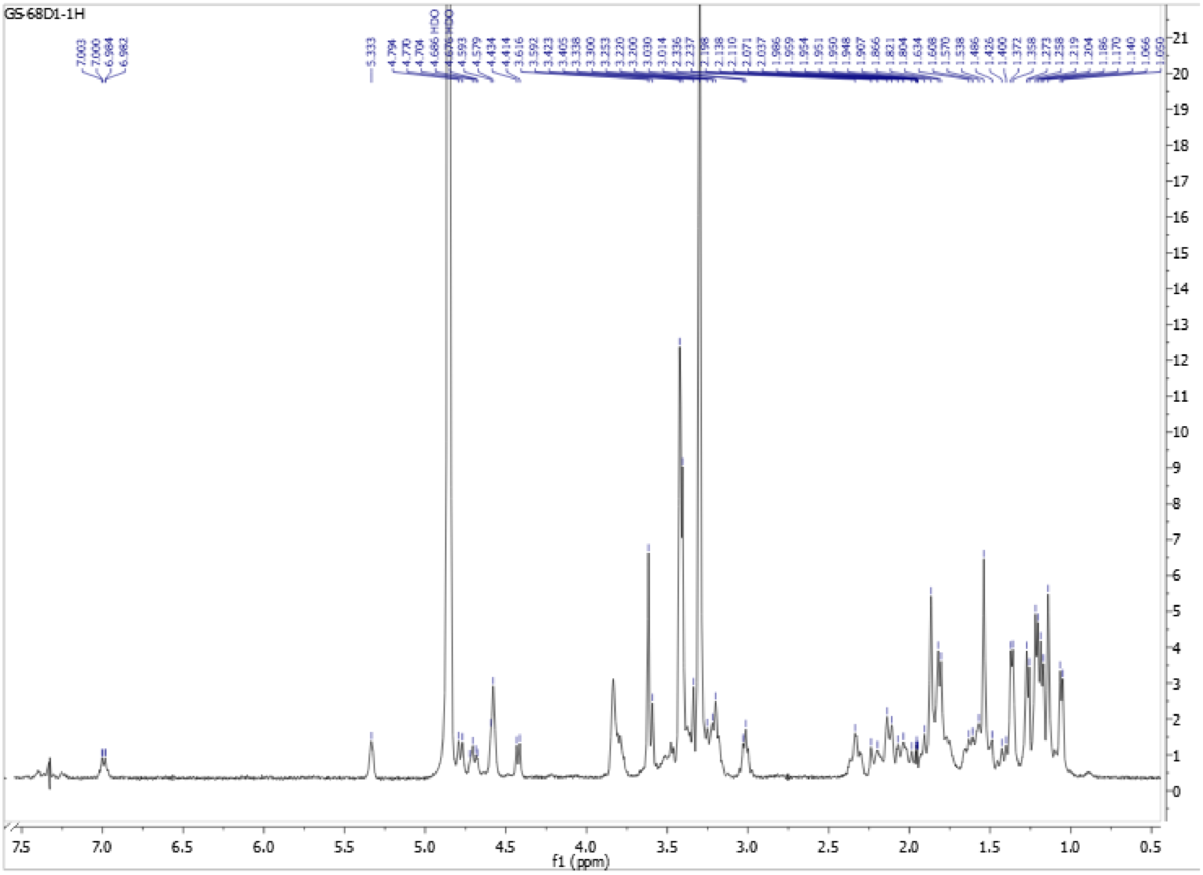

Figure S20: ^{13}C-NMR spectrum of compound **3**

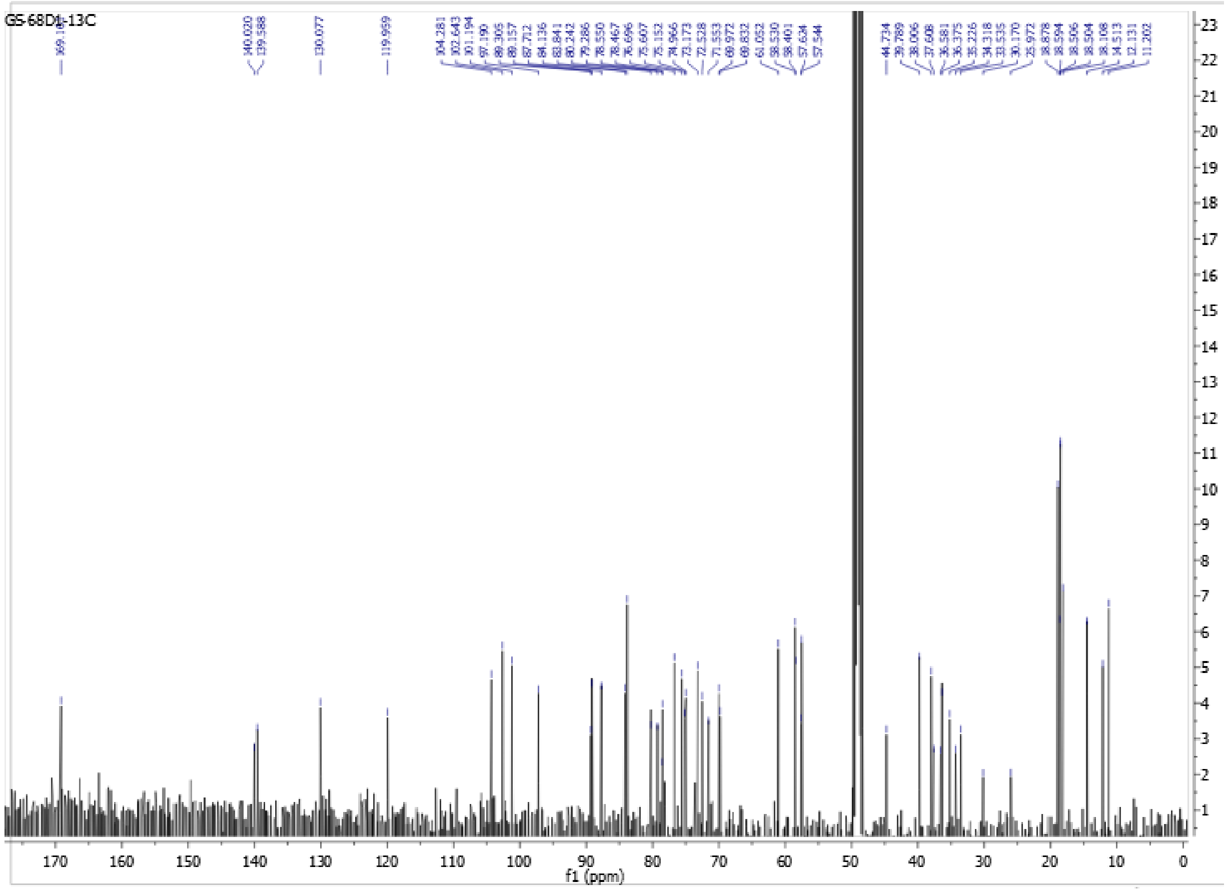

Figure S21: HSQC spectrum of compound **3**

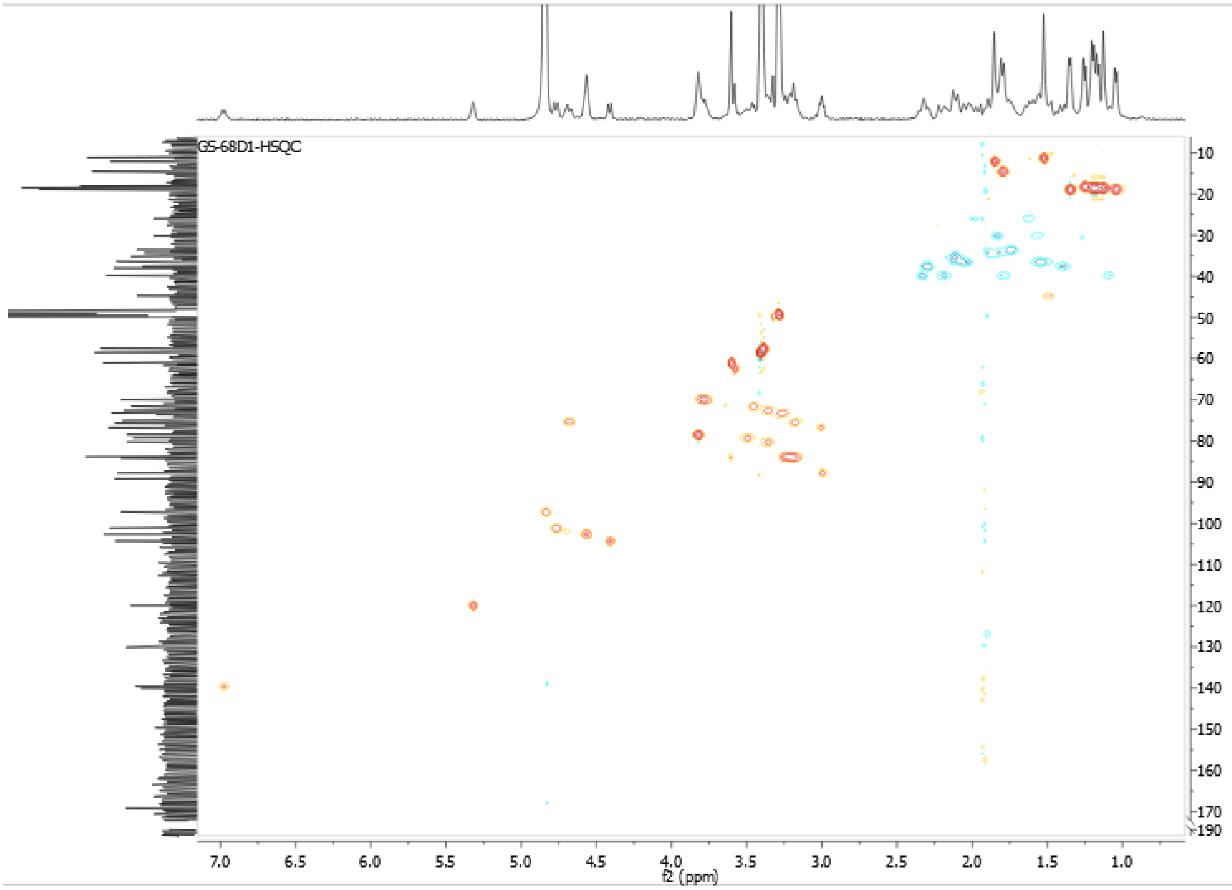

Figure S22: HMBC spectrum of compound **3**

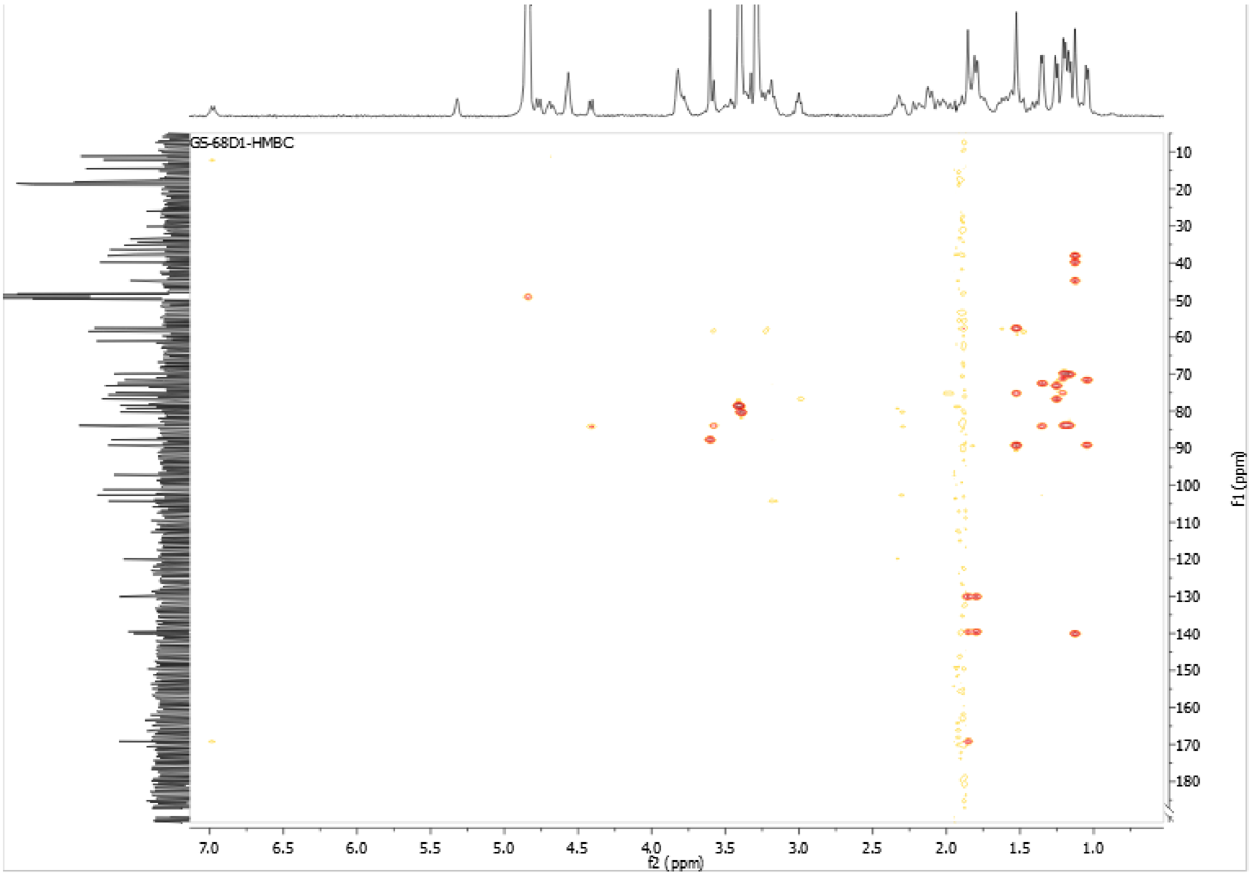

Figure S23: COSY spectrum of compound **3**

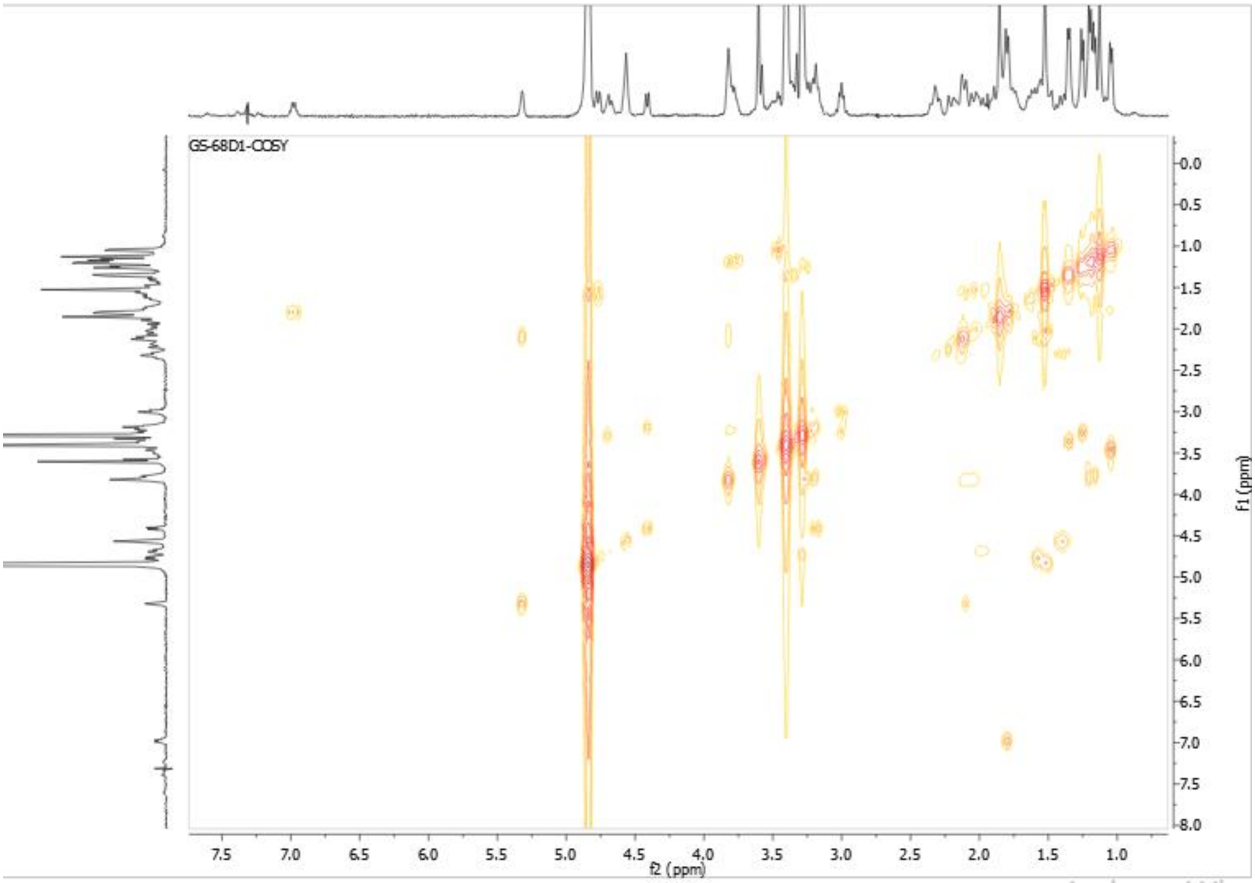

Figure S24: ROESY spectrum of compound **3**

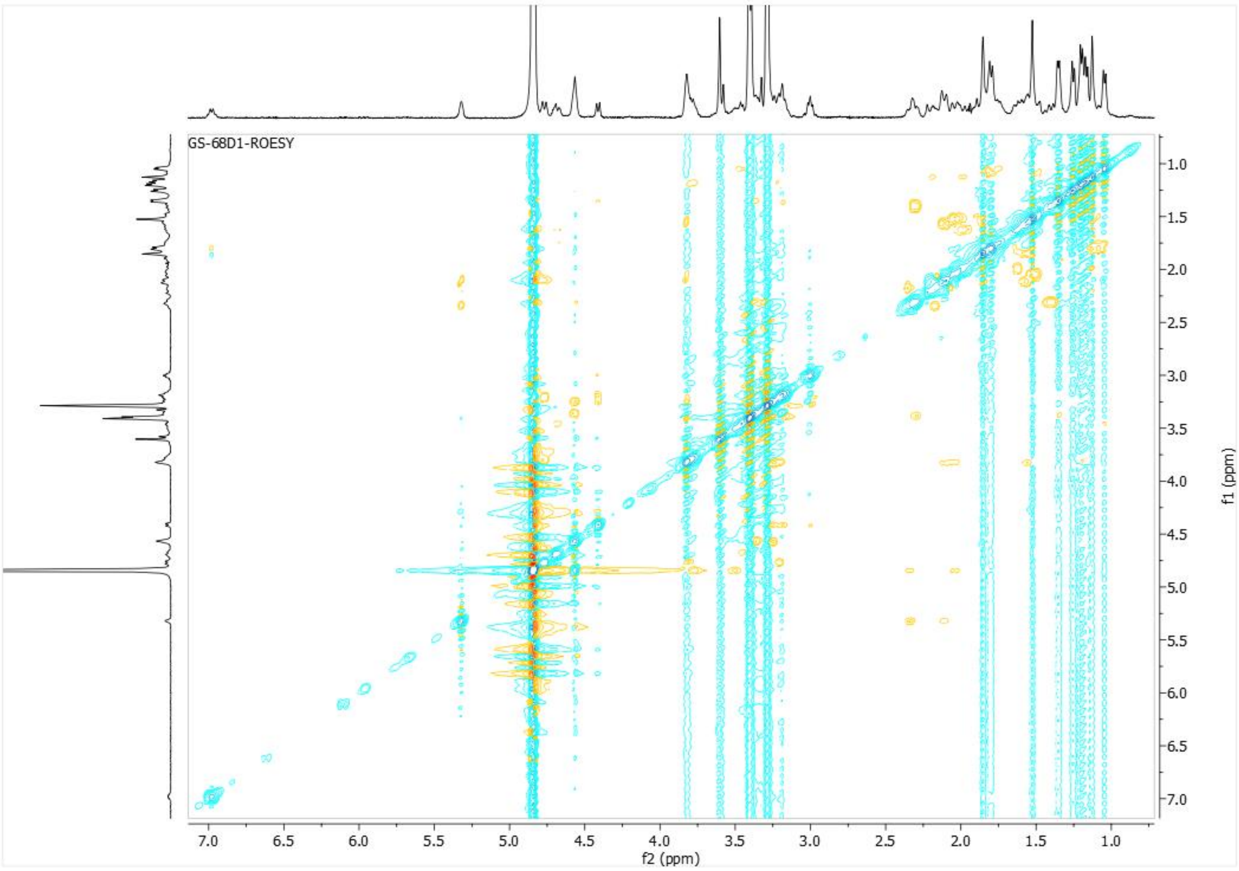

Figure S25: HR-ESI-MS of compound **4**

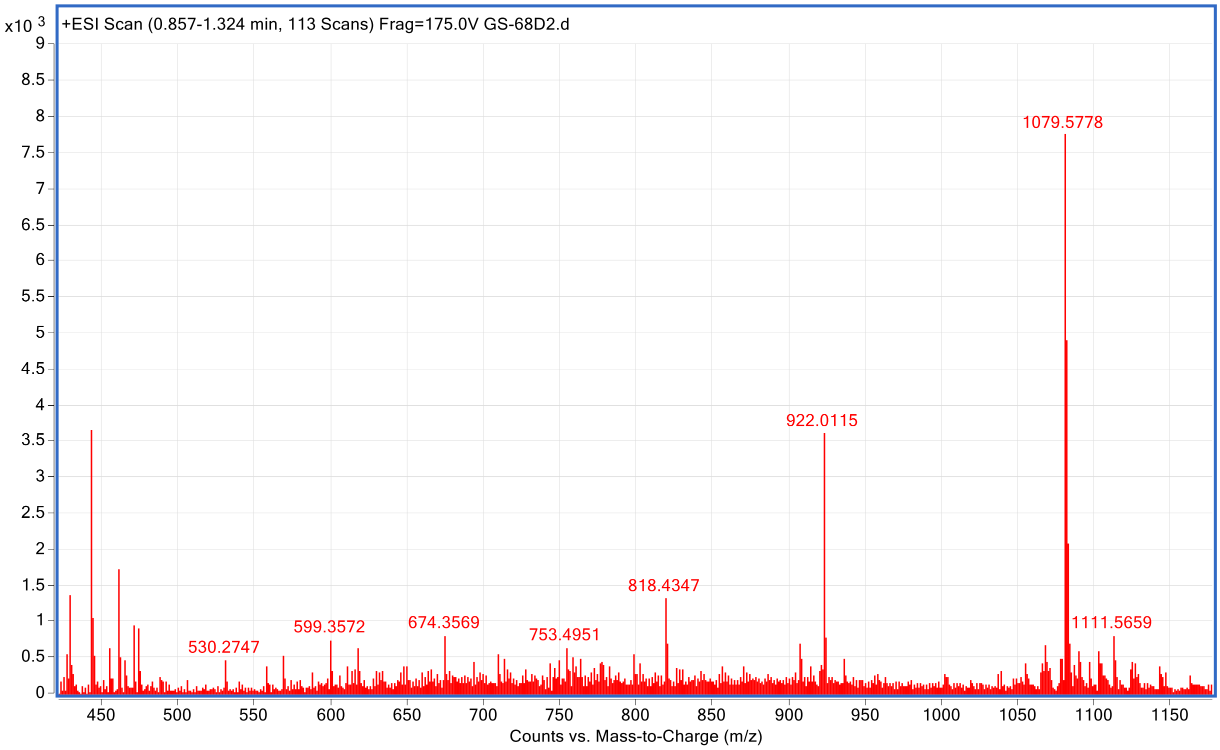

Figure S26: ^{1}H-NMR spectrum of compound **4**

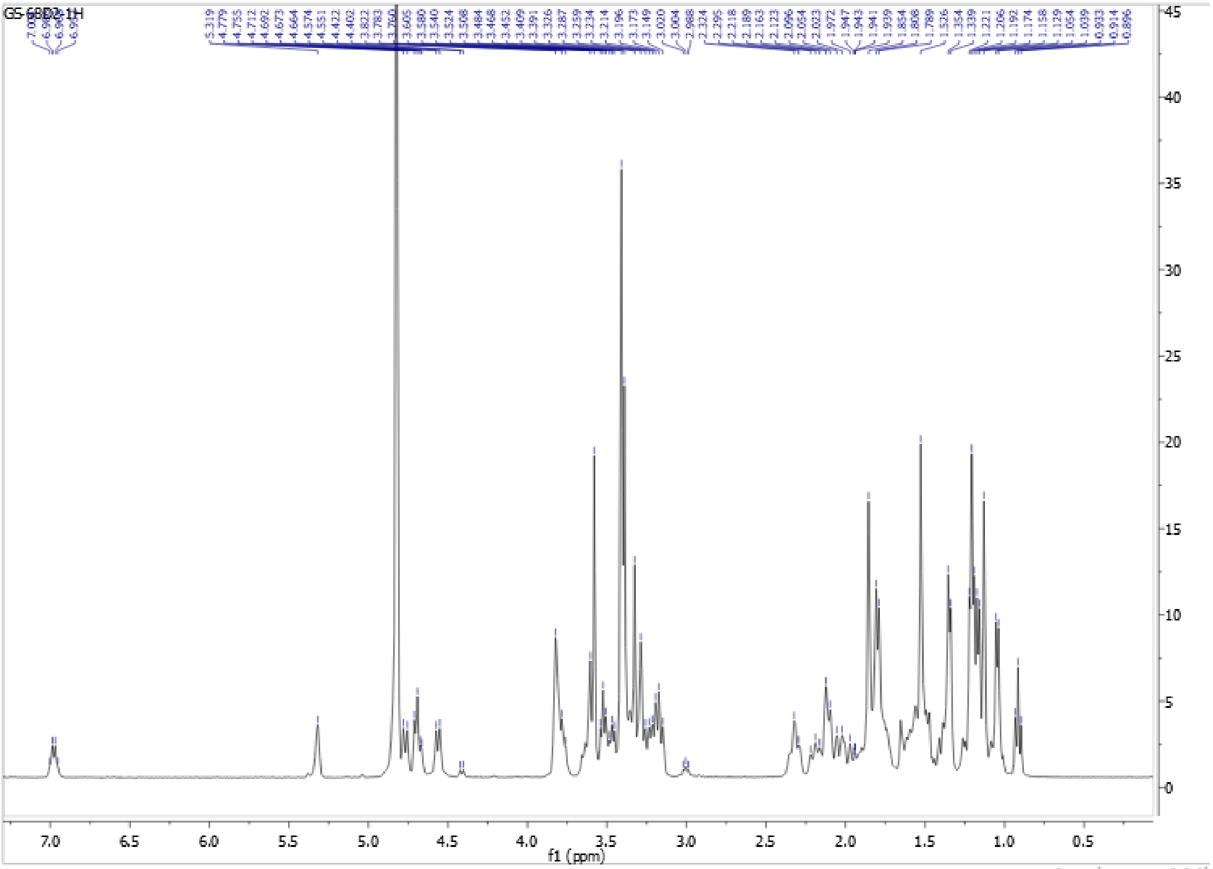

Figure S27: ^{13}C-NMR spectrum of compound **4**

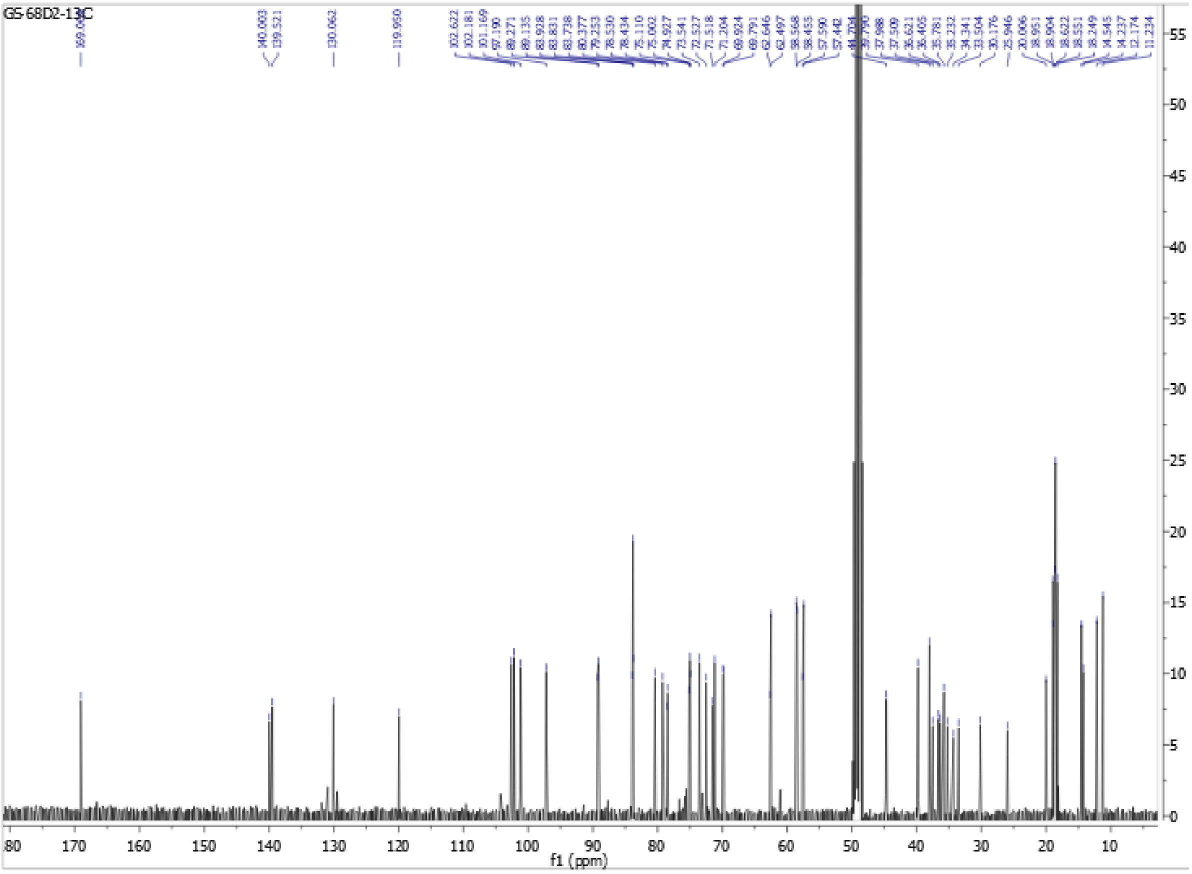

Figure S28: DEPT135 spectrum of compound **4**

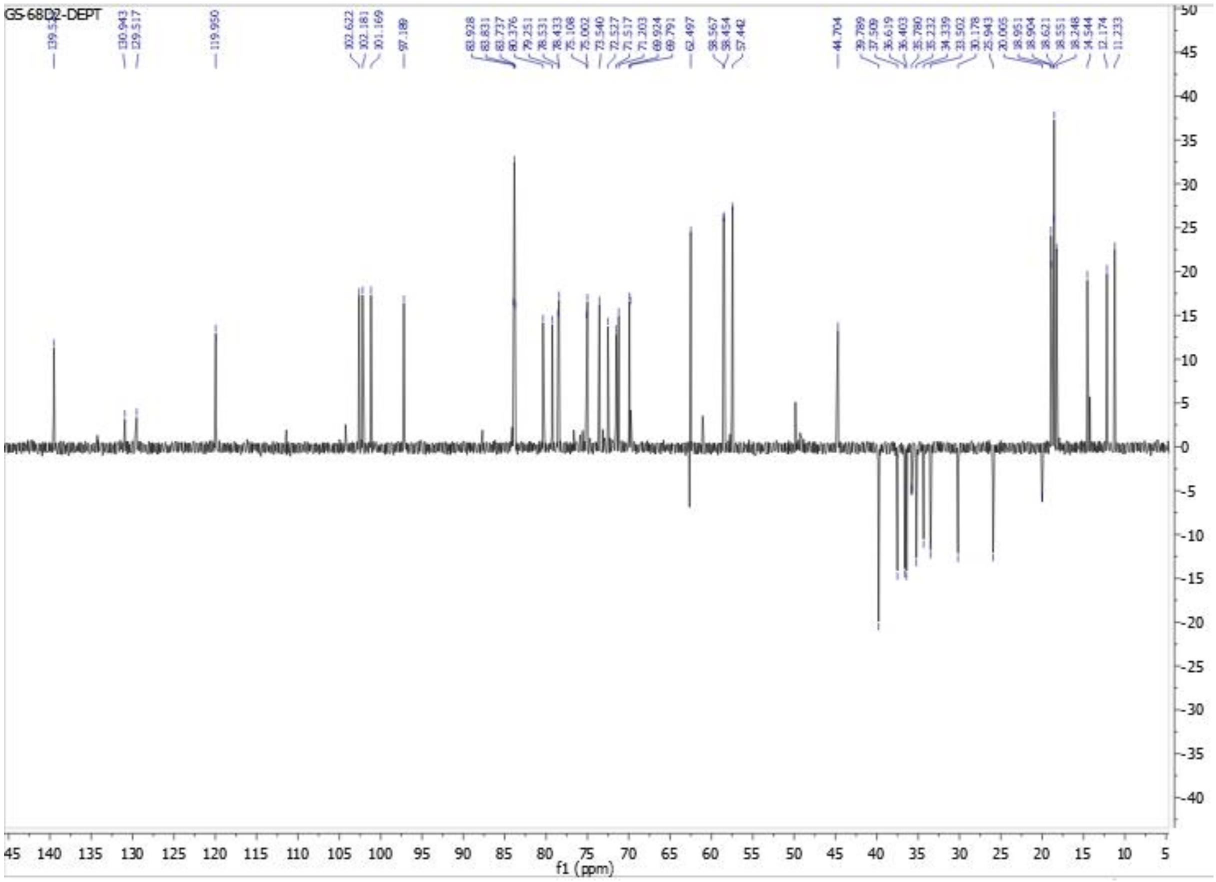

Figure S29: HSQC spectrum of compound **4**

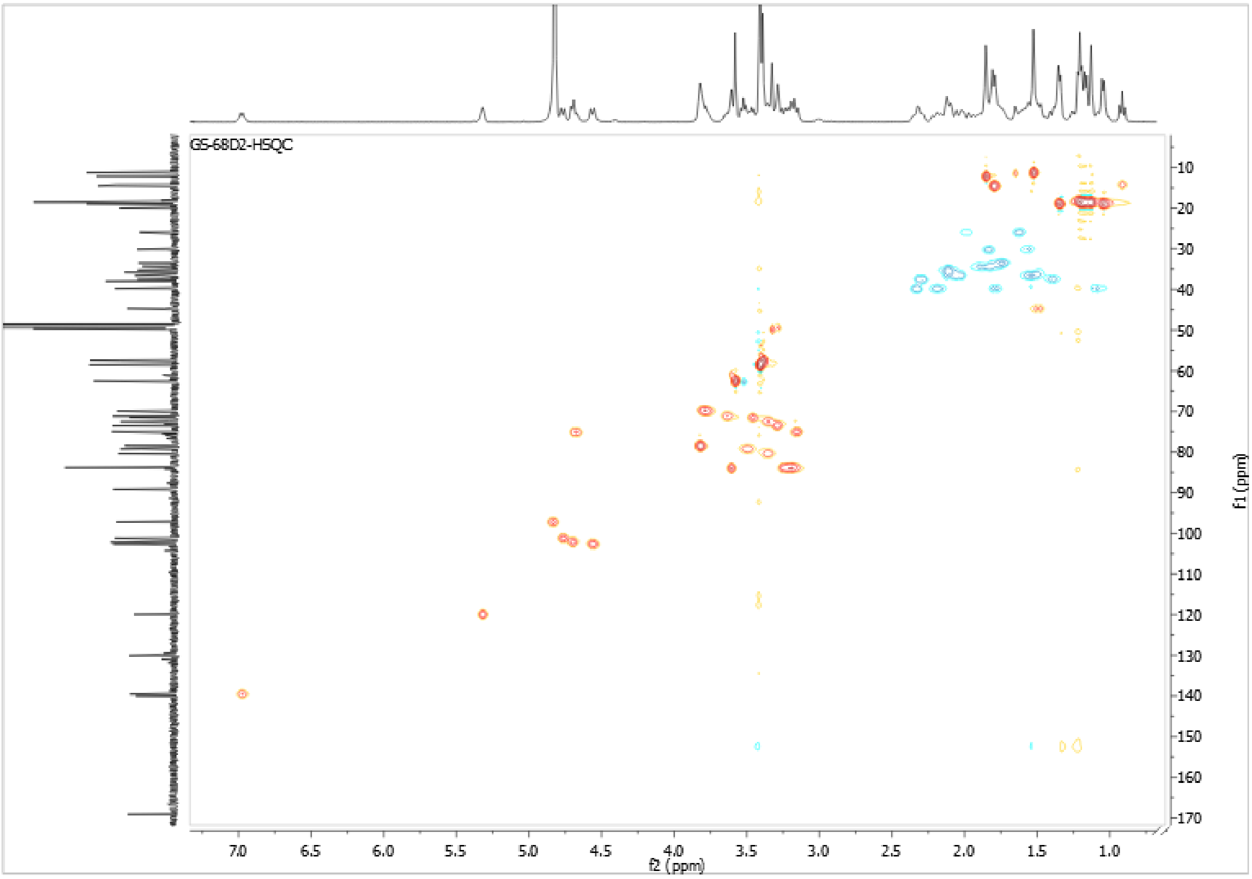

Figure S30: HMBC spectrum of compound **4**

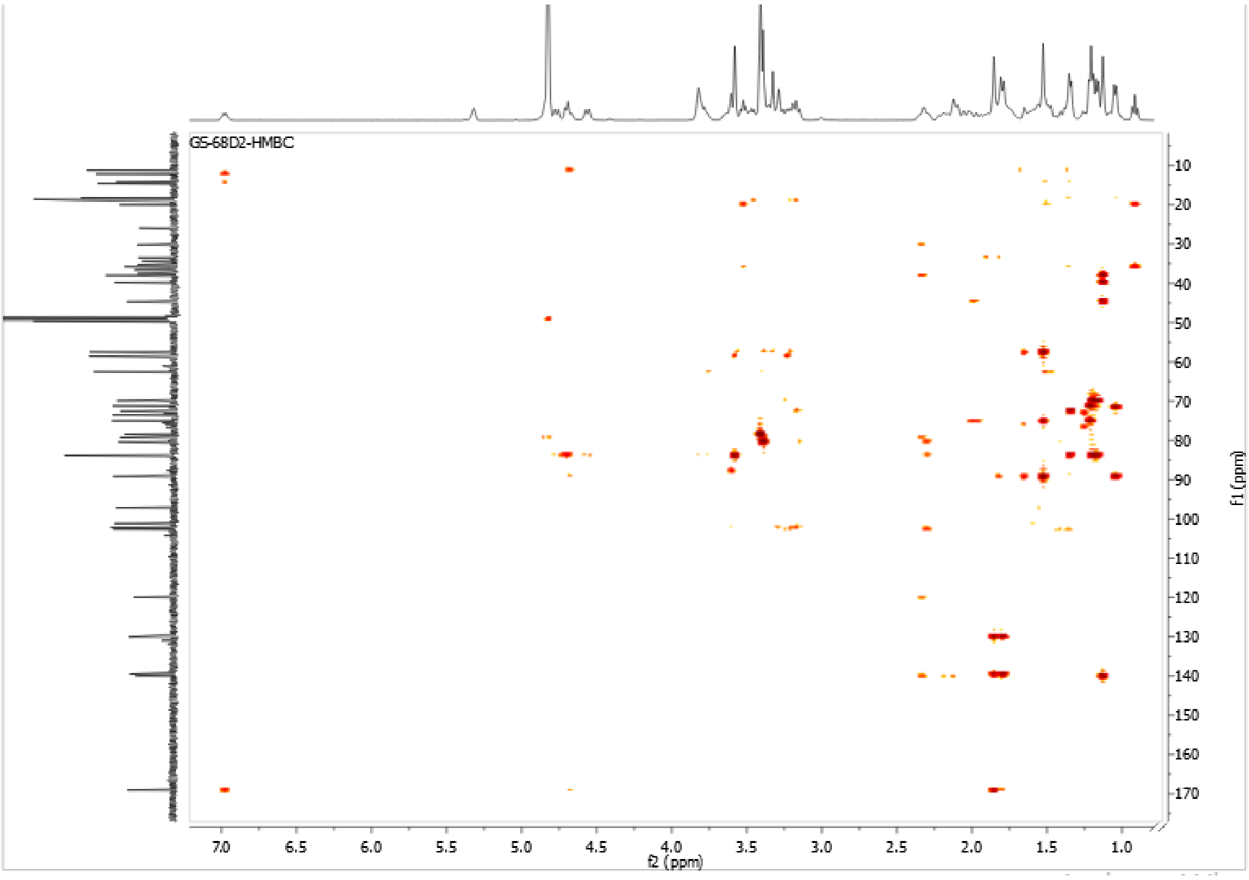

Figure S31: COSY spectrum of compound **4**

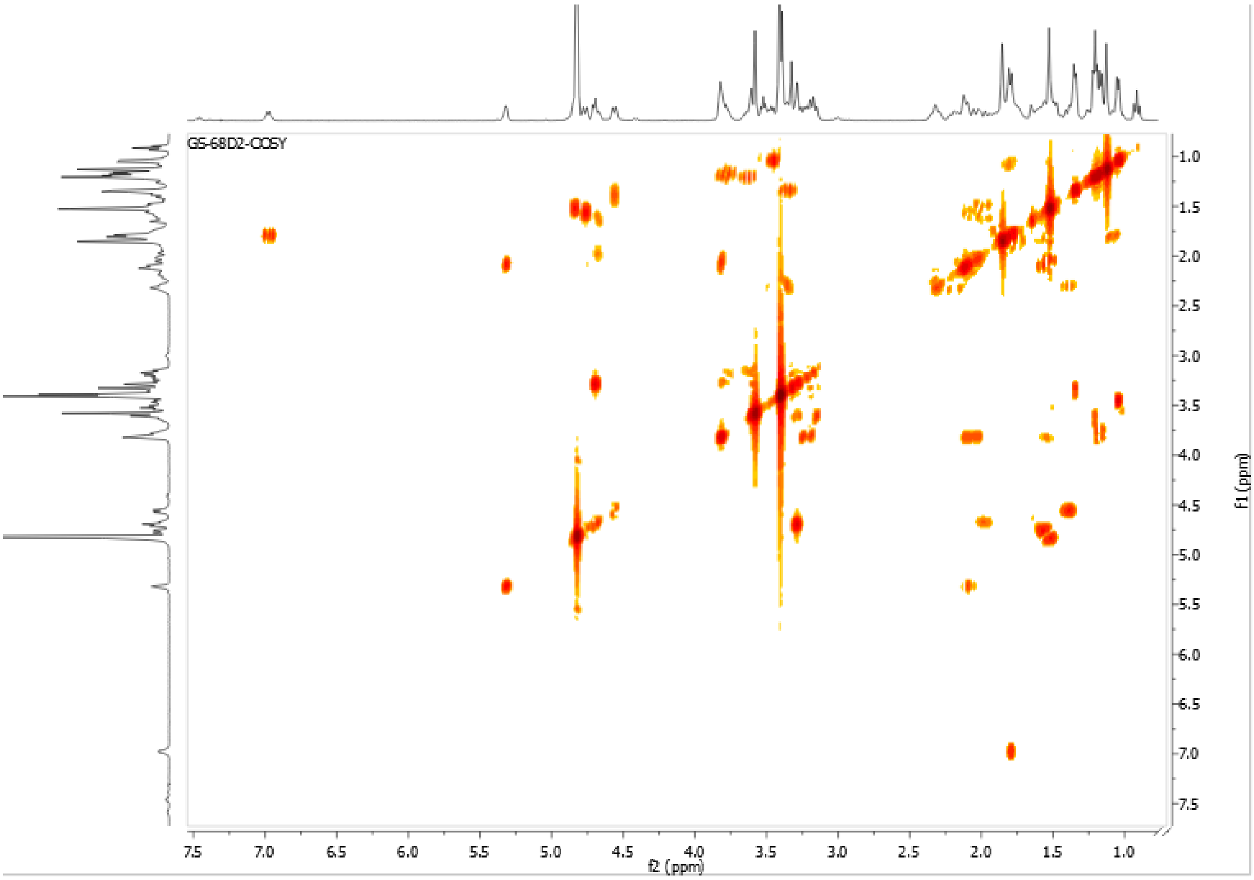

Figure S32: ROESY spectrum of compound **4**

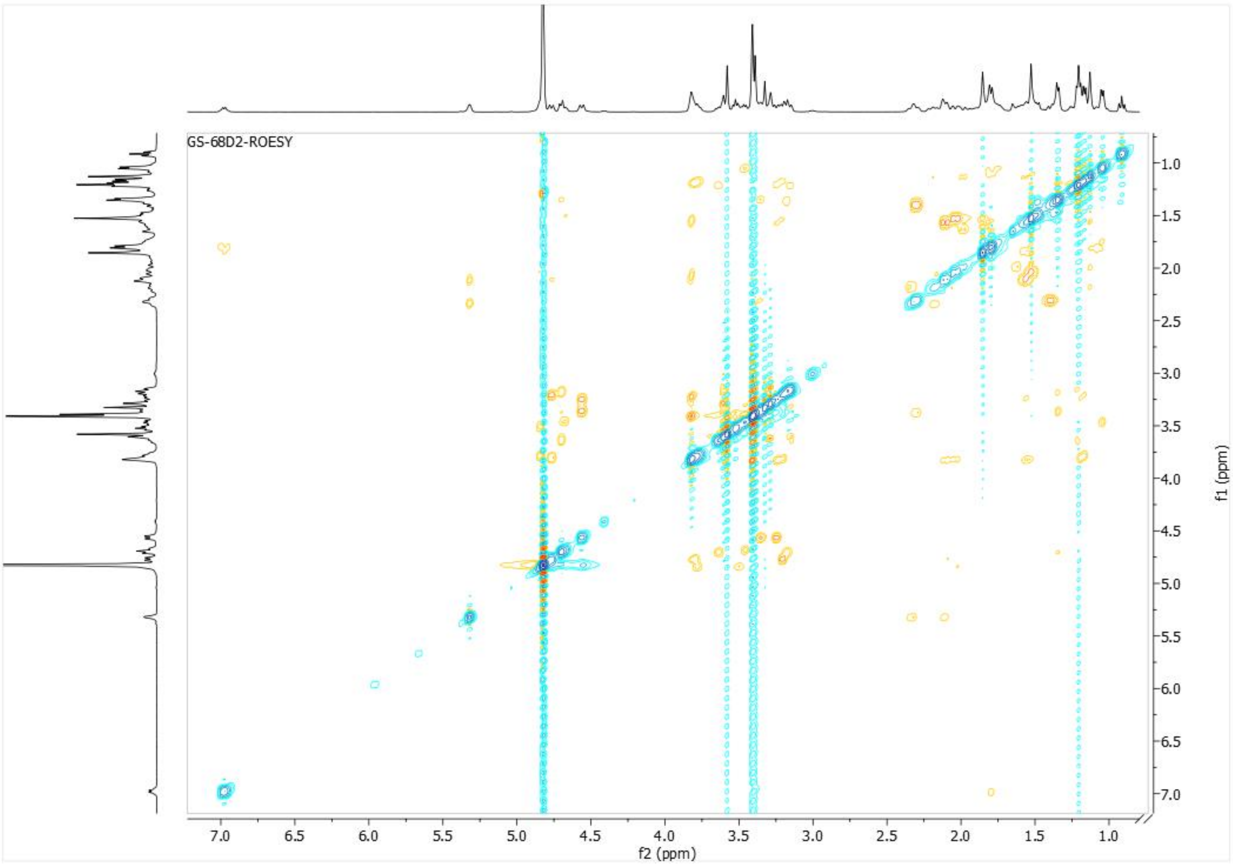

Figure S33: HR-ESI-MS of compound **5**

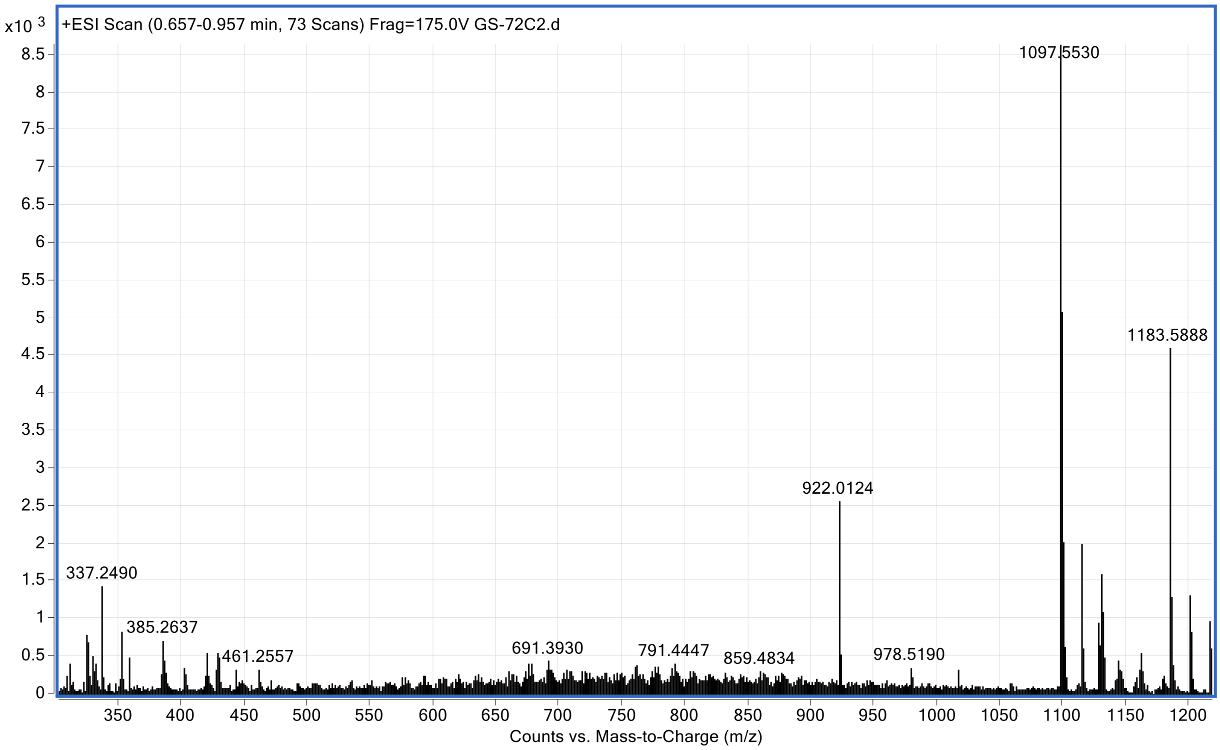

Figure S34: ^{1}H-NMR spectrum of compound **5**

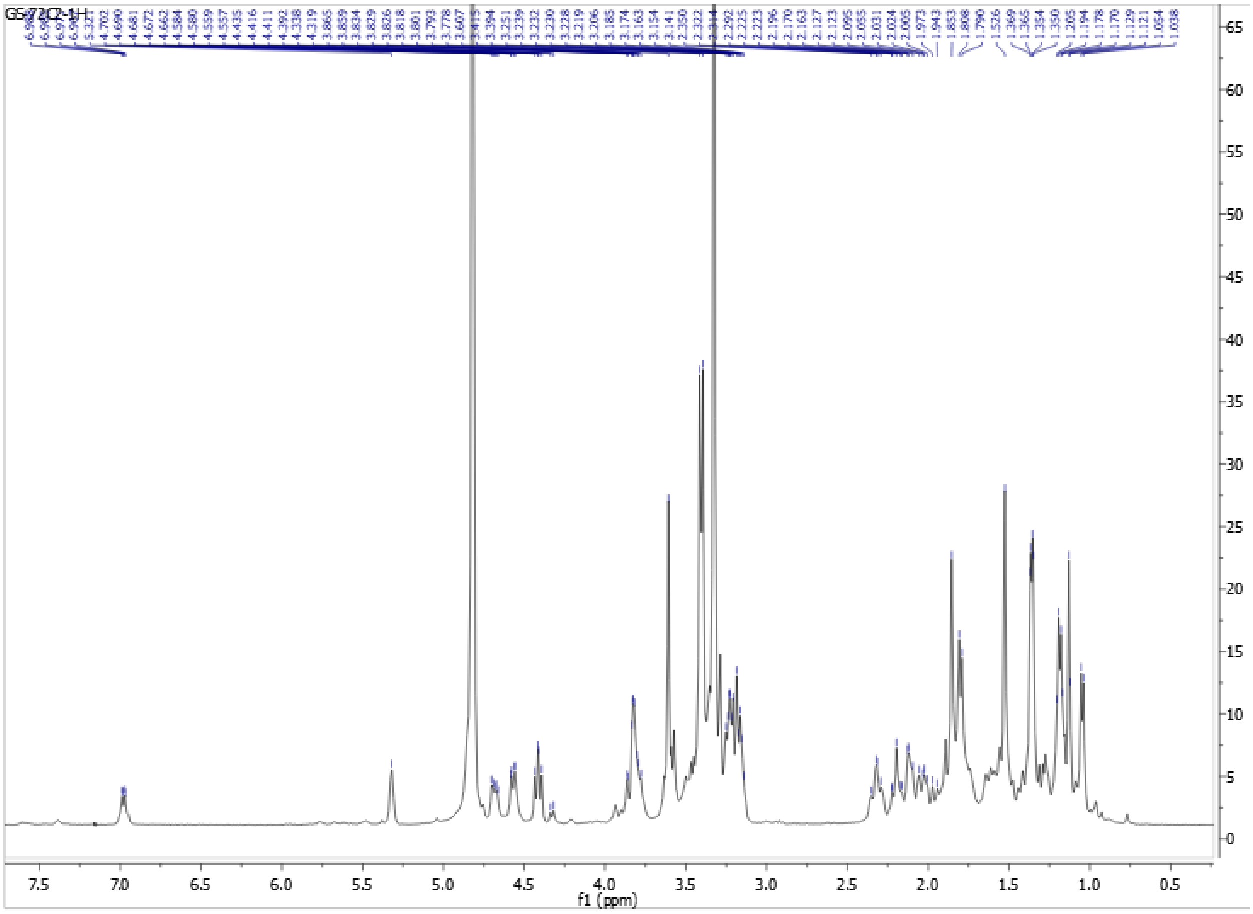

Figure S35: ^{13}C-NMR spectrum of compound **5**

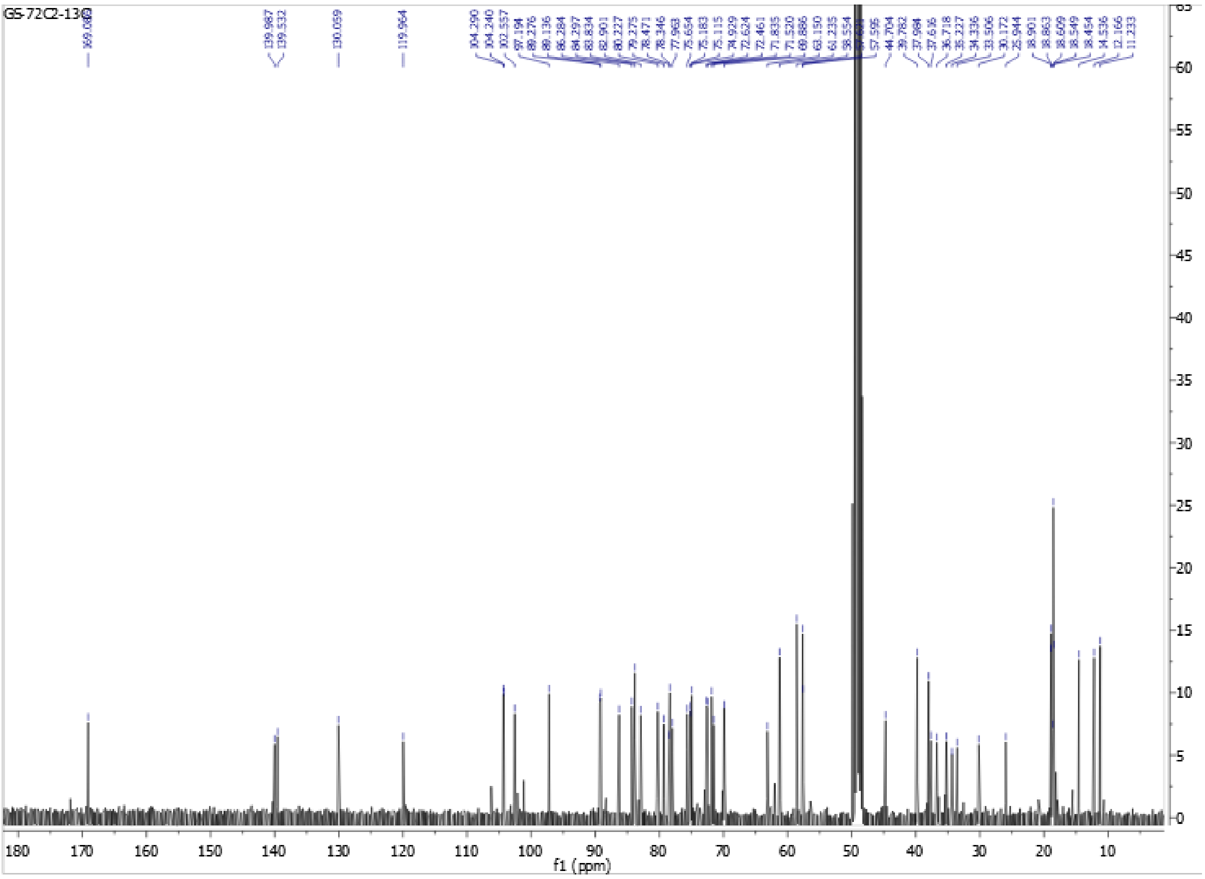

Figure S36: DEPT135 spectrum of compound **5**

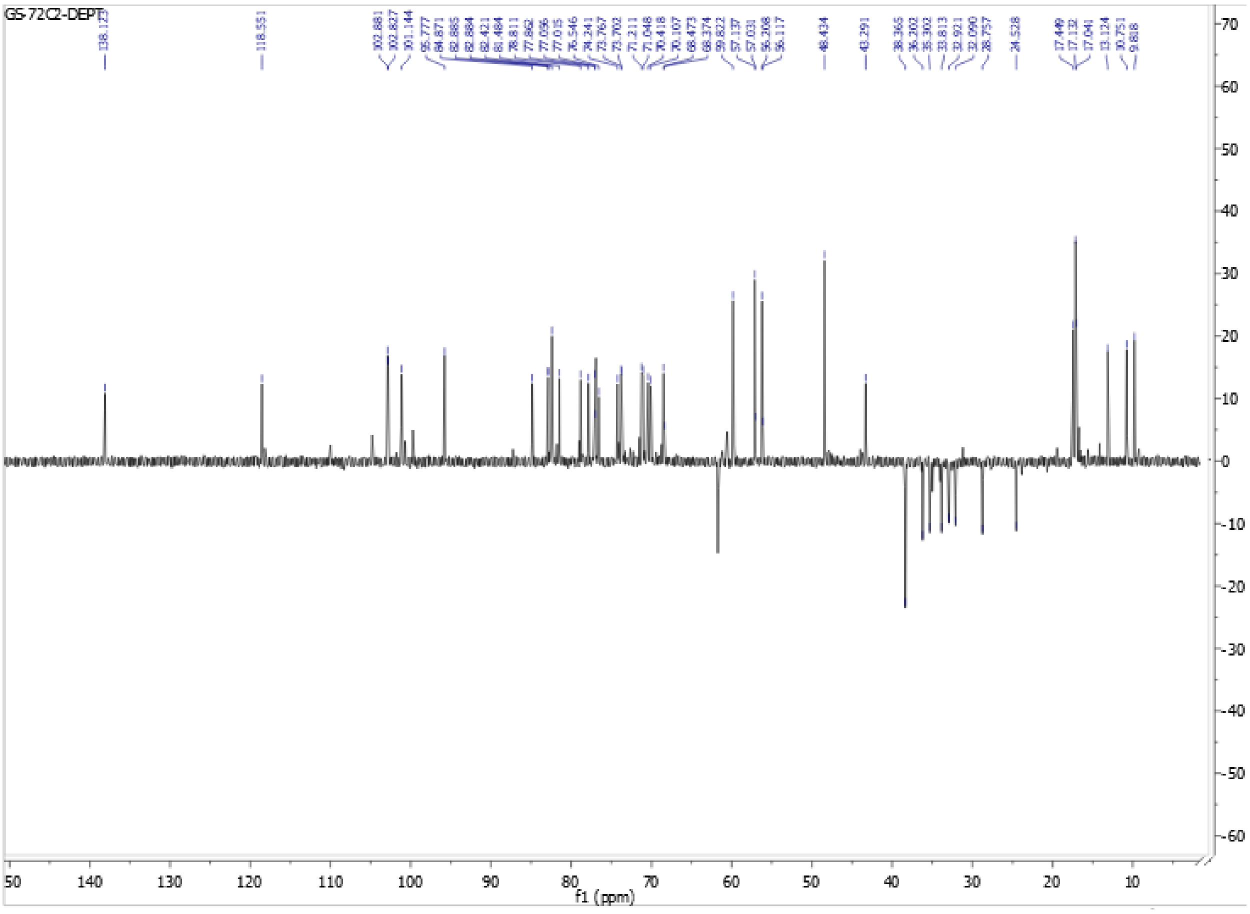

Figure S37: HSQC spectrum of compound **5**

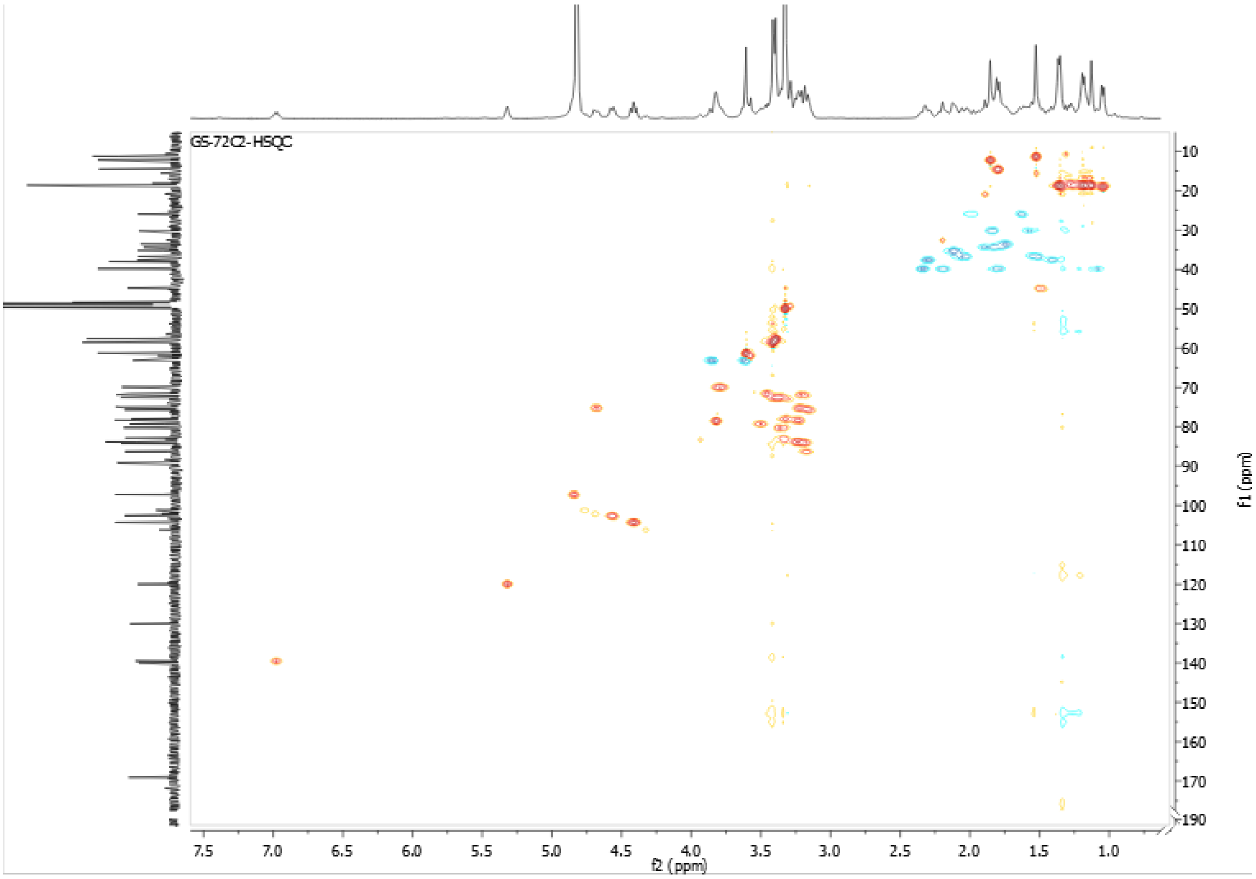

Figure S38: HMBC spectrum of compound **5**

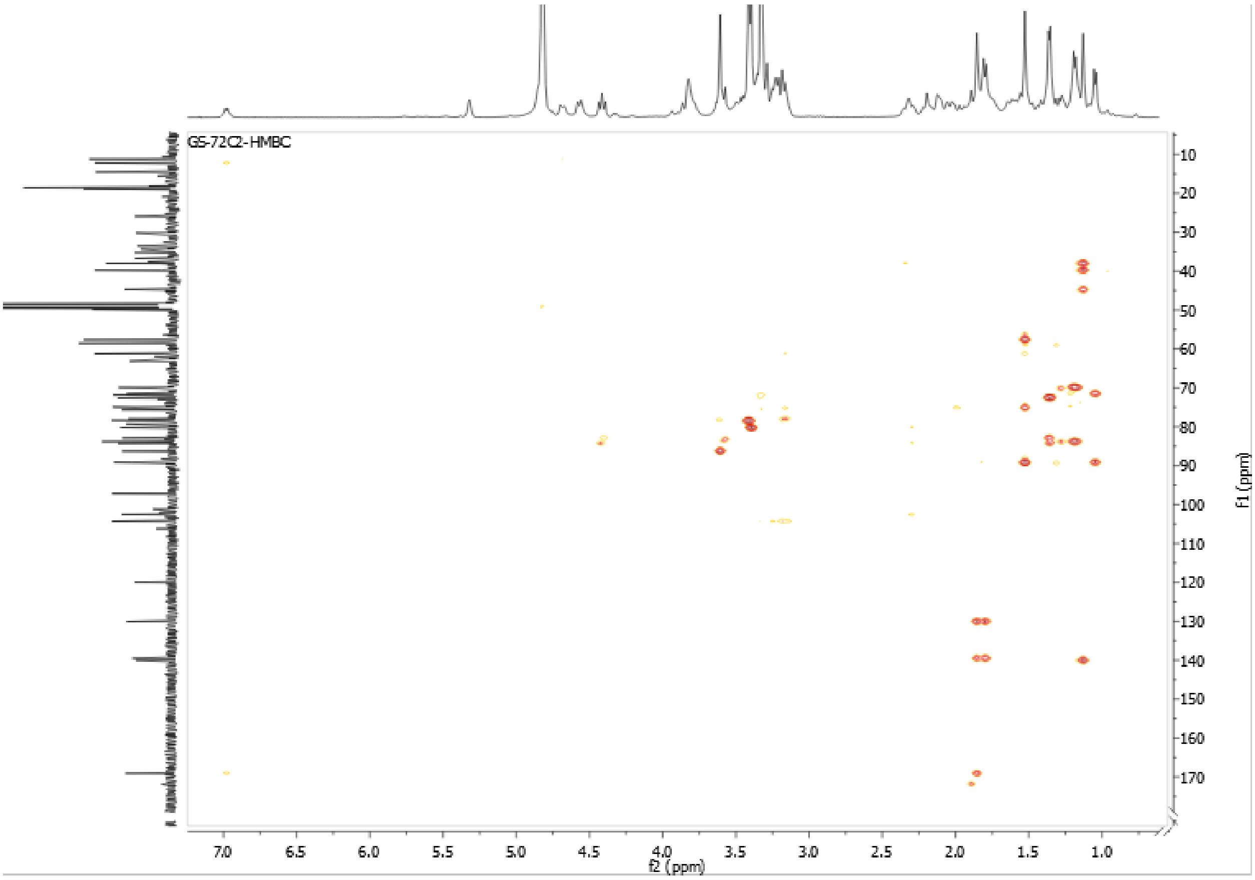

Figure S39: COSY spectrum of compound **5**

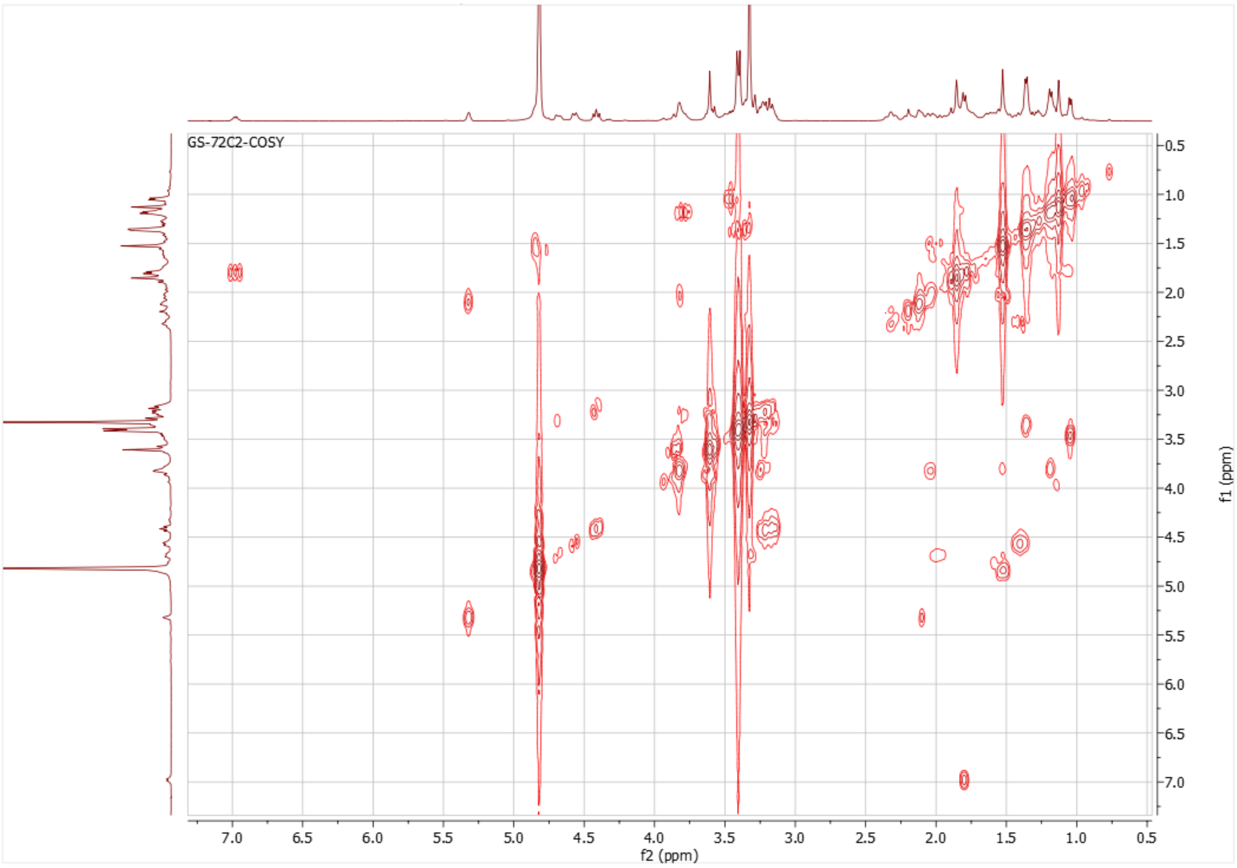

Figure S40: ^{1}H-NMR spectrum of compound **6**

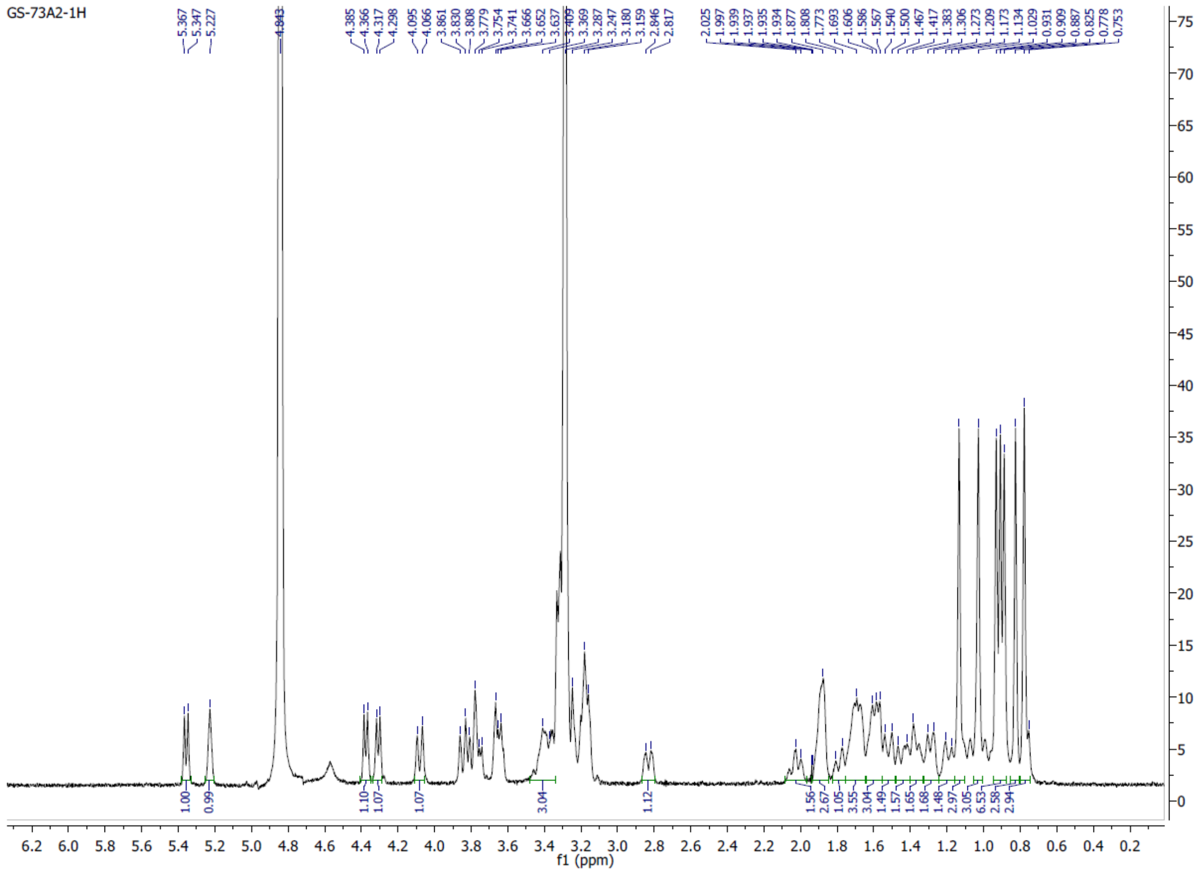

Figure S41: ^{13}C-NMR spectrum of compound **6**

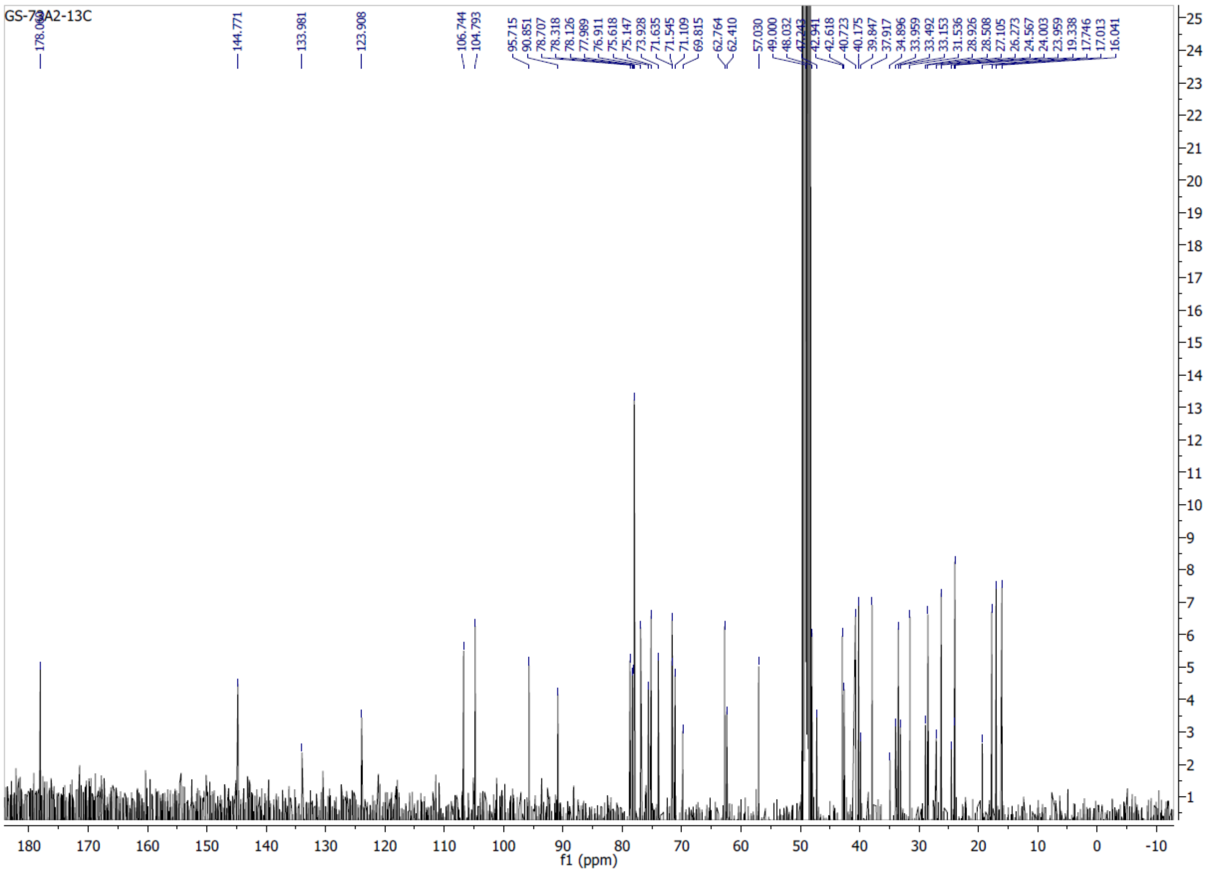

Figure S42: HSQC spectrum of compound **6**

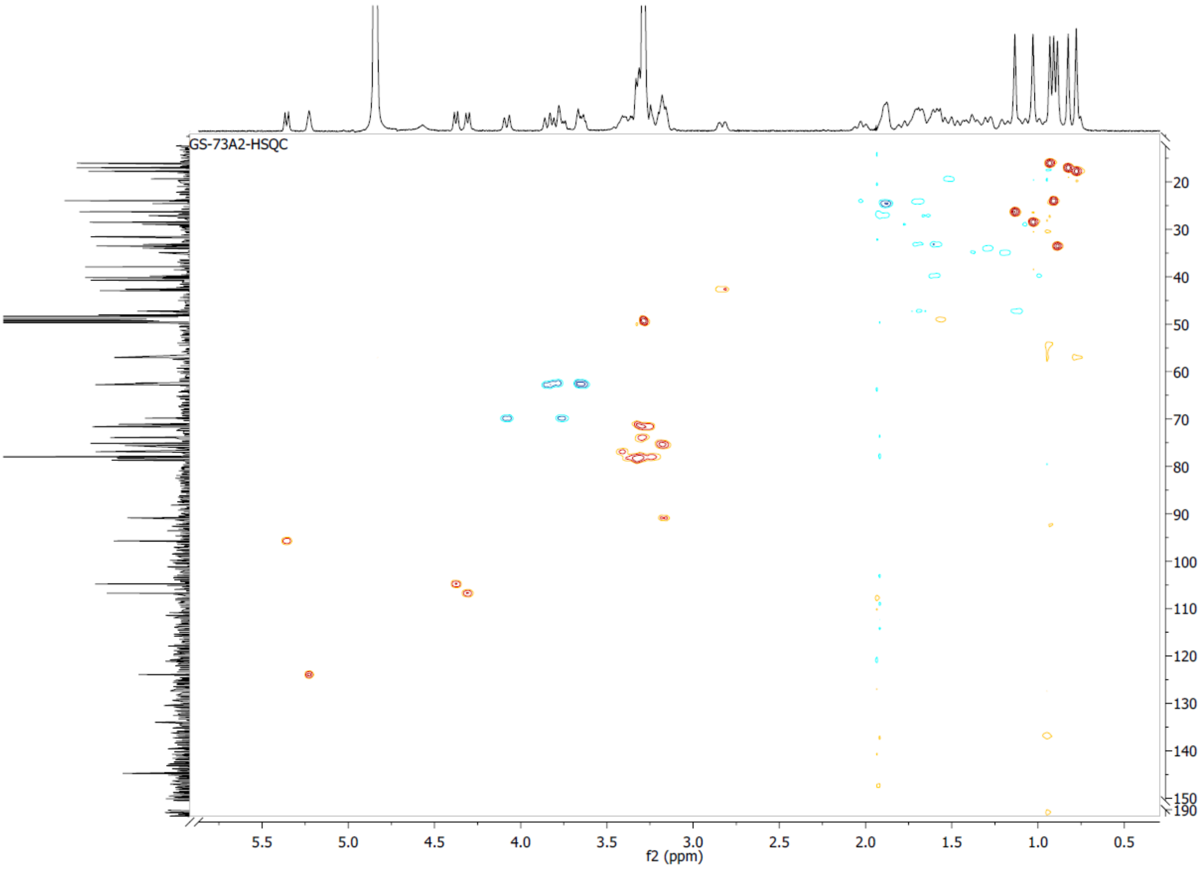

Figure S43: ^{1}H-NMR spectrum of compound **7**

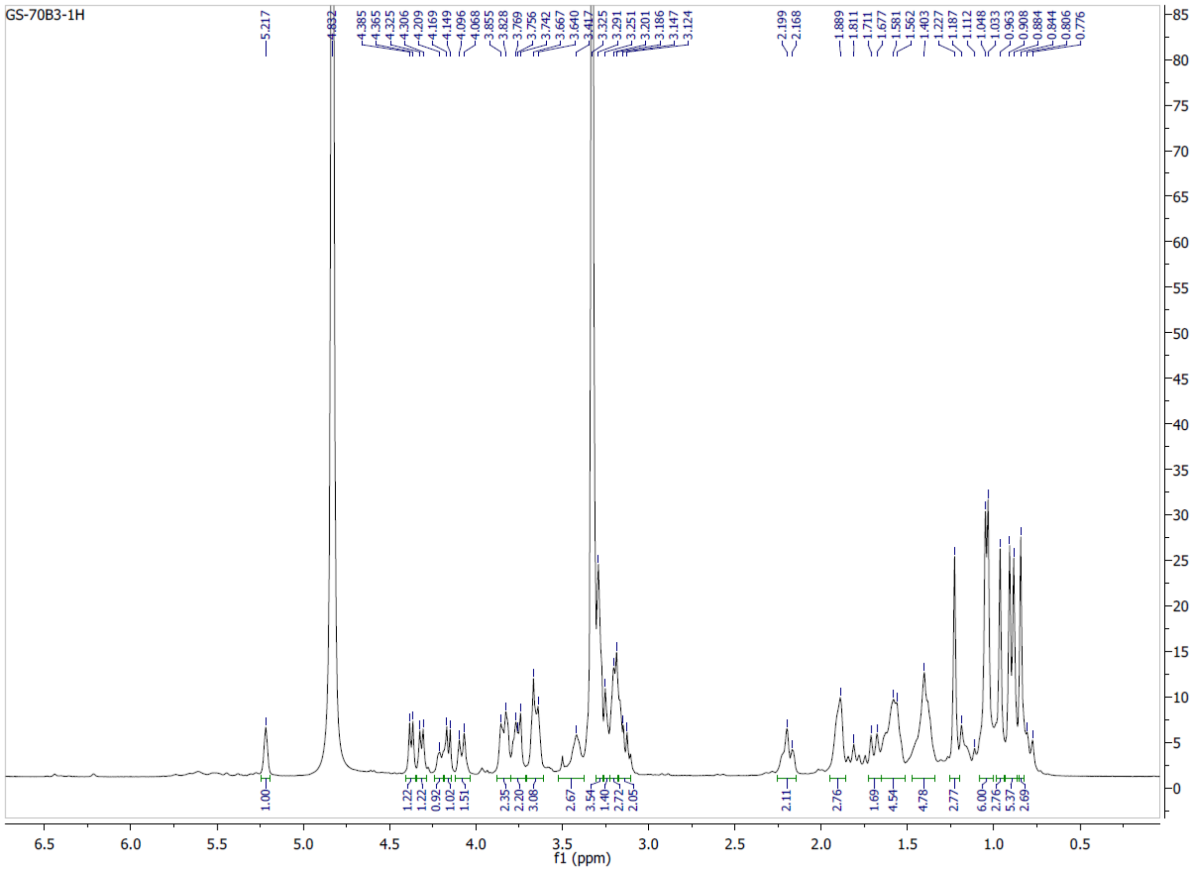

Figure S44: ^{13}C-NMR spectrum of compound **7**

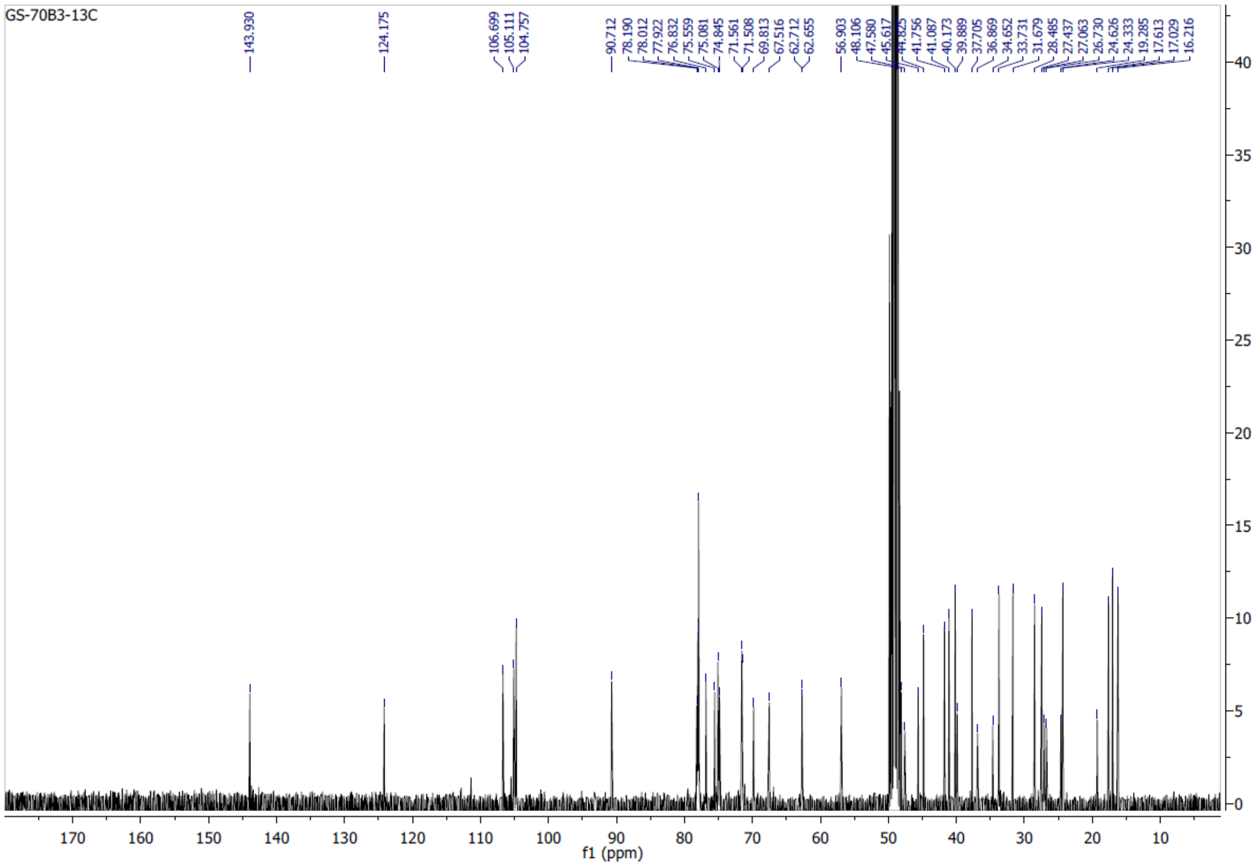

Figure S45: HSQC spectrum of compound **7**

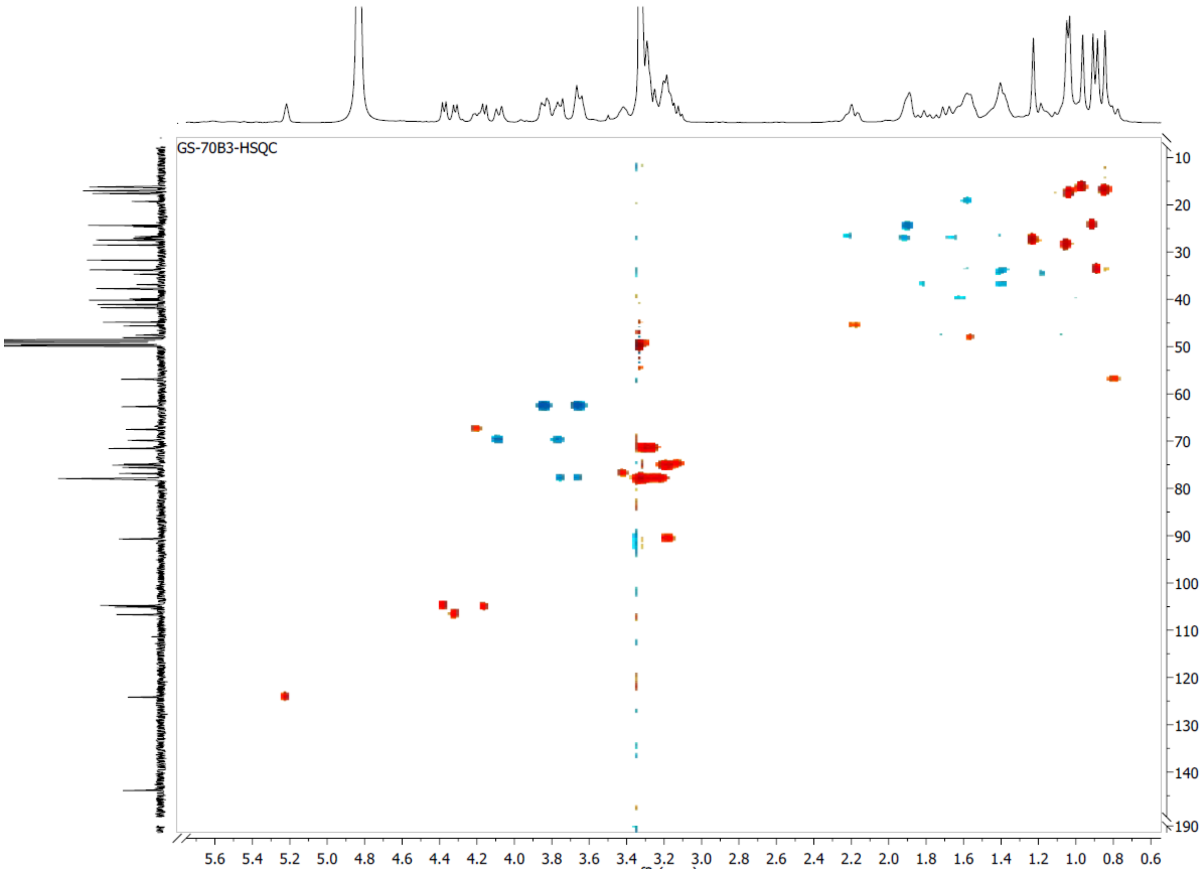

Figure S46: ^{1}H-NMR spectrum of compound **8**

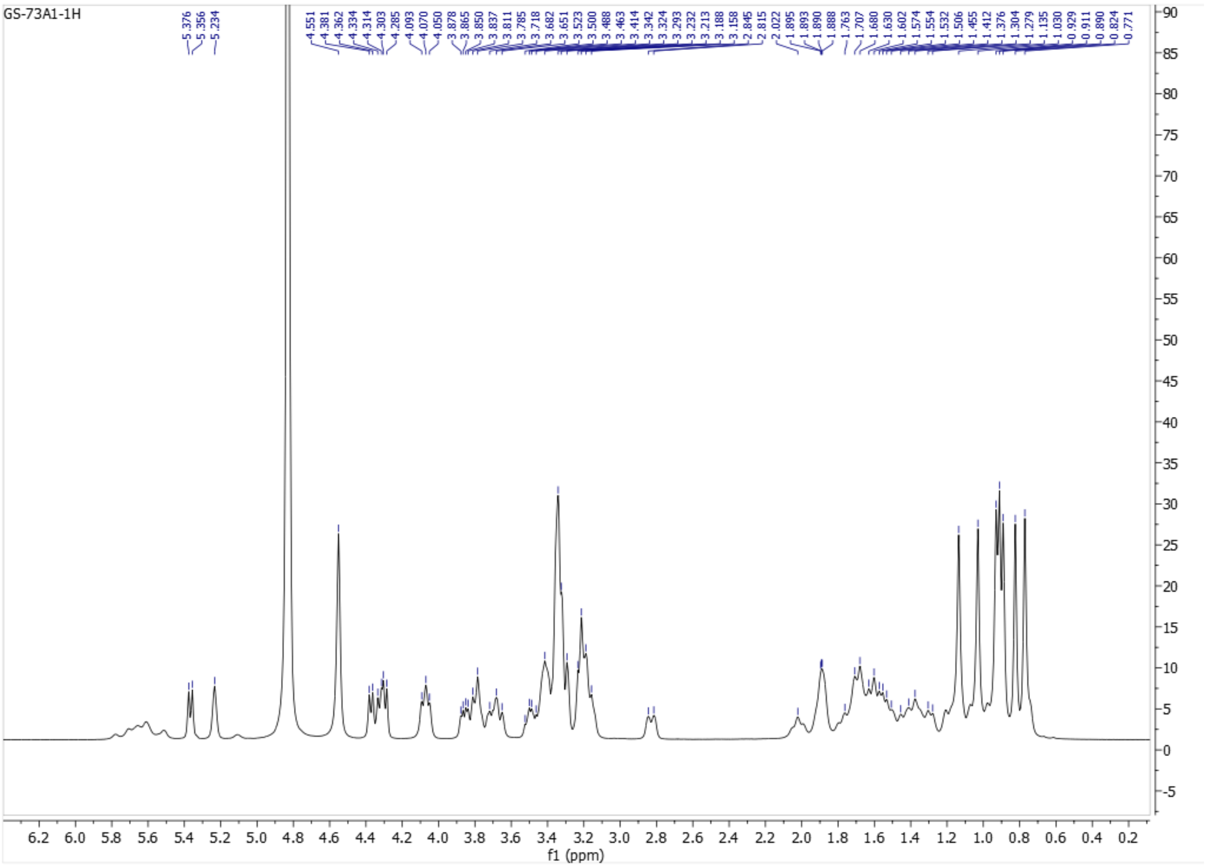

Figure S47: ^{13}C-NMR spectrum of compound **8**

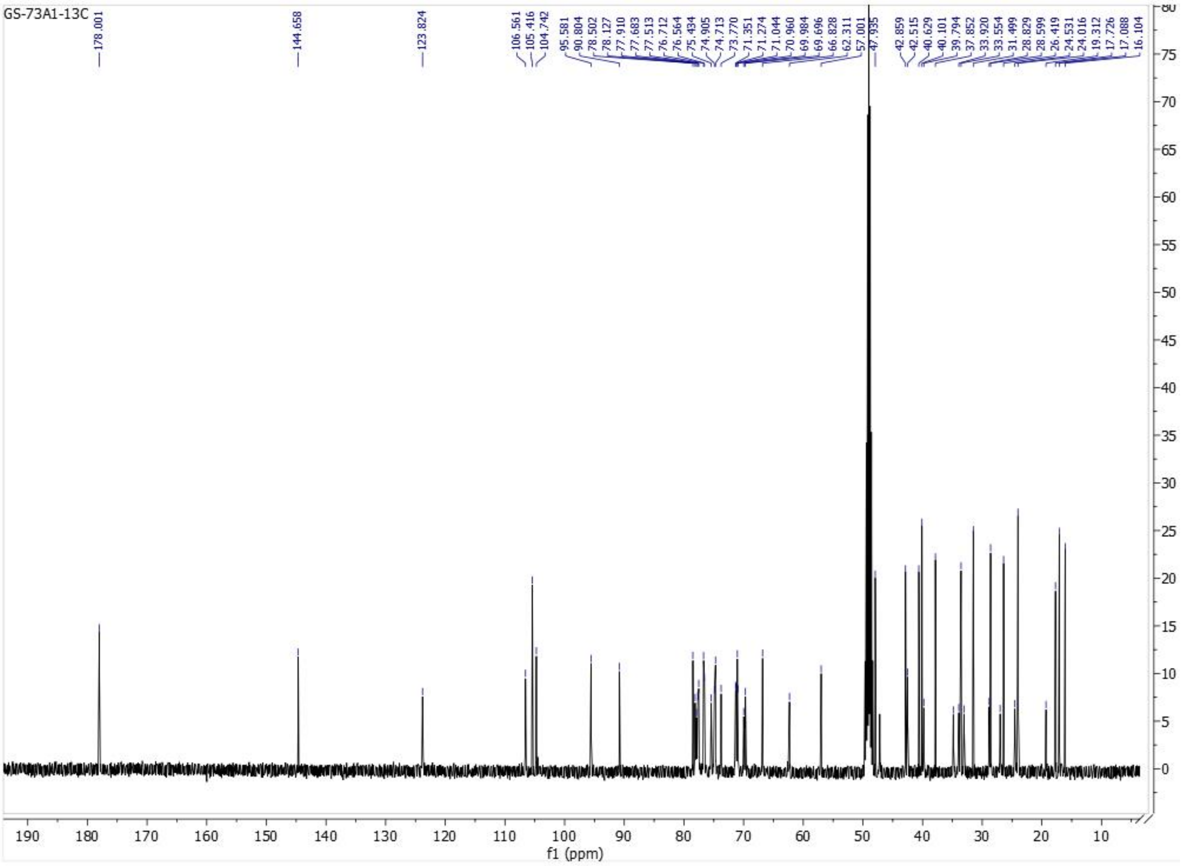

Figure S48: HSQC spectrum of compound **8**

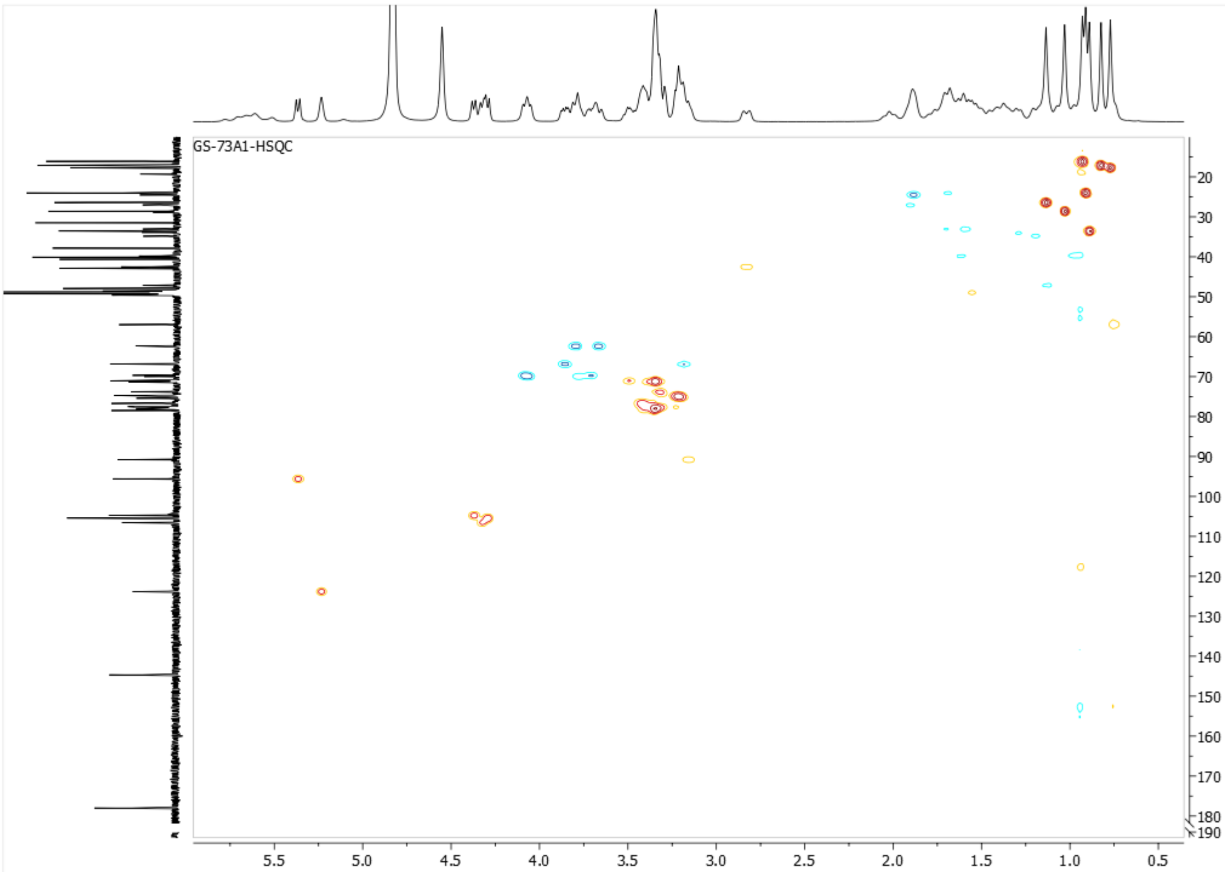

Figure S49: ^{1}H-NMR spectrum of compound **9**

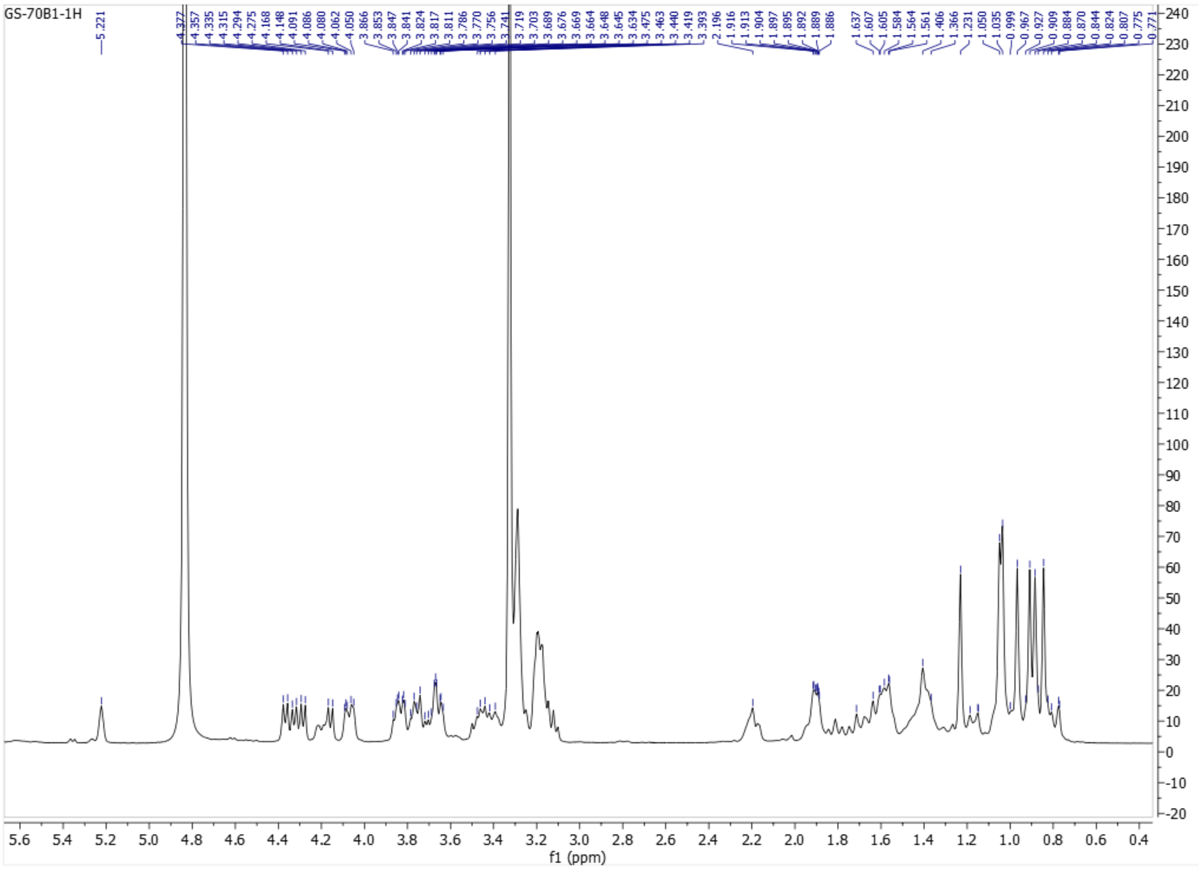

Figure S50: ^{13}C-NMR spectrum of compound **9**

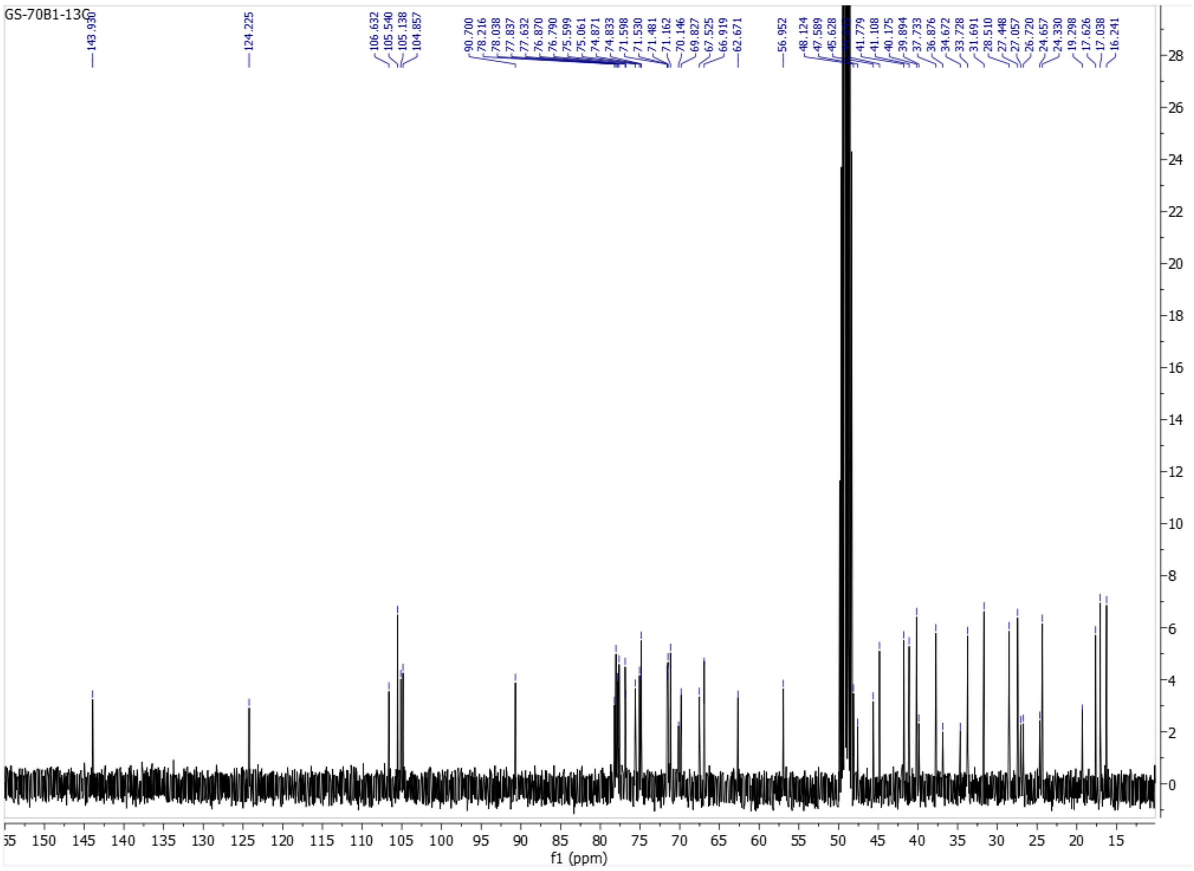

Figure S51: HSQC spectrum of compound **9**

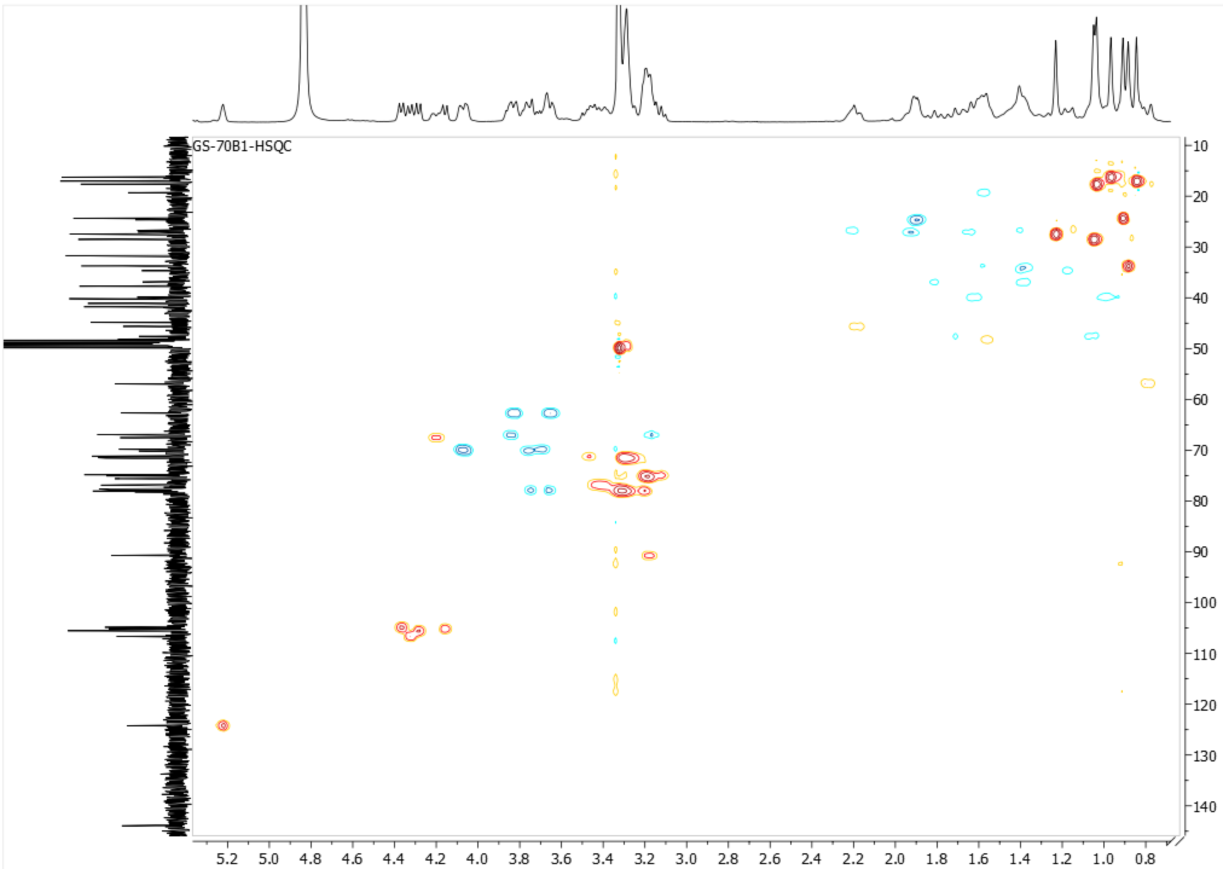

Supplement: Supplementary file 1 [file molecules-25-02525-s001.pdf]
